# Group Analysis in FieldTrip of Time-Frequency Responses: A Pipeline for Reproducibility at Every Step of Processing, Going From Individual Sensor Space Representations to an Across-Group Source Space Representation

**DOI:** 10.3389/fnins.2018.00261

**Published:** 2018-05-01

**Authors:** Lau M. Andersen

**Affiliations:** NatMEG, Department of Clinical Neuroscience, Karolinska Institutet, Stockholm, Sweden

**Keywords:** MEG, analysis pipeline, fieldtrip, beamformer, tactile expectations, group analysis, good practice

## Abstract

An important aim of an analysis pipeline for magnetoencephalographic (MEG) data is that it allows for the researcher spending maximal effort on making the statistical comparisons that will answer his or her questions. The example question being answered here is whether the so-called beta rebound differs between novel and repeated stimulations. Two analyses are presented: going from individual sensor space representations to, respectively, an across-group sensor space representation and an across-group source space representation. The data analyzed are neural responses to tactile stimulations of the right index finger in a group of 20 healthy participants acquired from an Elekta Neuromag System. The processing steps covered for the first analysis are MaxFiltering the raw data, defining, preprocessing and epoching the data, cleaning the data, finding and removing independent components related to eye blinks, eye movements and heart beats, calculating participants' individual evoked responses by averaging over epoched data and subsequently removing the average response from single epochs, calculating a time-frequency representation and baselining it with non-stimulation trials and finally calculating a grand average, an across-group sensor space representation. The second analysis starts from the grand average sensor space representation and after identification of the beta rebound the neural origin is imaged using beamformer source reconstruction. This analysis covers reading in co-registered magnetic resonance images, segmenting the data, creating a volume conductor, creating a forward model, cutting out MEG data of interest in the time and frequency domains, getting Fourier transforms and estimating source activity with a beamformer model where power is expressed relative to MEG data measured during periods of non-stimulation. Finally, morphing the source estimates onto a common template and performing group-level statistics on the data are covered. Functions for saving relevant figures in an automated and structured manner are also included. The protocol presented here can be applied to any research protocol where the emphasis is on source reconstruction of induced responses where the underlying sources are not coherent.

## Introduction

Magnetoencephalography (MEG) studies often include questions about how different experimental factors relate to brain activity. To test experimental factors, one can create contrasting conditions to single out the unique contributions of each experimental factor. Single subject studies using MEG would face two limitations in singling out the contributions of experimental factors. Firstly, the MEG signals of interest are mostly too weak to find due to the noise always present in MEG data, and secondly there is an interest in making an inference from one's data to the population as a whole. Group level analyses can circumvent these limitations by increasing the signal-to-noise ratio and by allowing for an inference to the population as a whole. It should be mentioned though that single subject analyses can be meaningful for clinicians trying to diagnose patients. Epilepsy investigations are routinely carried out on single subjects. Despite the fact that most studies rely on group level comparisons to increase the signal-to-noise ratio and for allowing for inferences to the population, almost all tutorials are based on single subject analyses. In the current paper, part of a special issue devoted to group analysis pipelines, I try to remedy this for anyone fancying using the FieldTrip (Oostenveld et al., 2011) analysis package. The data is structured according to the Magnetoencephalography Brain Imaging Data structure (MEG-BIDS) format to ease access to the data (Galan et al., 2017) and it is only dependent on having access to MATLAB (MathWorks: mathworks.com).

The basic idea of the current group pipeline is to set up a structure that allows for:

- Running group analysis at the channel and source levels
- Dividing output files into folders belonging to the respective subjects and recordings
- Applying an operation across a group of subjects
- (Re)starting the analysis at any intermediate point by saving output for each intermediate point
- Plotting the results in a way that allows for changing the figures in a principled, but flexible manner

A structure that allows for all four points will minimize the time that researchers have to spend on (1) double-checking that the right input goes into the right functions; (2) making sure that output and intermediate steps can be accessed meaningfully; (3) applying operations efficiently across groups of subjects; (4) re-processing data if changes to any intermediate step are desirable.

### The neuroscientific experiment

Since the focus is on how to conduct a group analysis, the neuroscientific questions answered with the pipeline are not novel. The focus is rather on the pipeline facilitating other experimenters' research, so that they efficiently can answer their own novel and interesting questions. Specifically, the pipeline will be centered around reconstructing induced activity using a beamformer approach. Induced activity is activity that is not phase-locked to a given event, say the stimulation of the finger, but which is related to the event in terms of timing and frequency. For example, the presentation of a stimulus may consistently be followed by an increase of the power of, say, the 10 Hz part of the power spectrum. Because this increase is not phase-locked to the event it would averaged away in a classical evoked analysis, where time-courses are averaged together (Gröchenig, 2013). Using a beamforming approach the origin of the induced responses can be localized (Hillebrand and Barnes, 2005; Hillebrand et al., 2005). Similar approaches have been used successfully to localize induced responses in the visual domain (Muthukumaraswamy and Singh, 2013), induced responses in the sensory-motor domain (Jurkiewicz et al., 2006), induced responses in the auditory domain (Weisz et al., 2014), induced responses related to attentional recruitment (Dalal et al., 2009; Ishii et al., 2014), induced responses related to face processing (Luo et al., 2007), induced responses related to the so-called resting state network (Hillebrand et al., 2012), induced responses related to working memory (van Dijk et al., 2010), induced responses related to mismatch detection (Garrido et al., 2015) and many more. Thus, the pipeline presented is based on a robust and well-tested procedure.

The reserved digital object identifier for the data repository, where data for this experiment and scripts for the pipeline can be freely downloaded is: doi: 10.5281/zenodo.998518. The corresponding URL is: https://zenodo.org/record/998518. The study that the data are taken from is not printed yet. The updated github code can be found at https://github.com/ualsbombe/omission_frontiers.

## Materials and equipment

### Subjects

Twenty participants volunteered to take part in the experiment (eight males, 12 females, Mean Age: 28.7 y; Minimum Age: 21; Maximum Age: 47). The experiment was approved by the local ethics committee, Regionala etikprövningsnämnden i Stockholm. Both written and oral informed consent were obtained from all subjects.

### Paradigm

**Figure 1:**
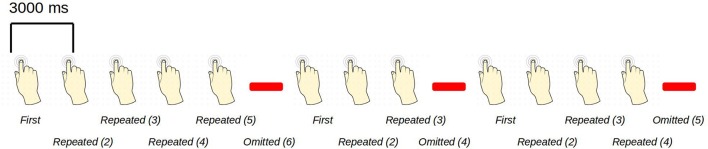
An example sequence of the experimental paradigm is shown. The annotations on the bottom show the coding used throughout for the different events of interest. Stimulations happened at a regular pace, every 3 s. When omissions occurred, there were thus 6 s between two consecutive stimulations.

The paradigm is based on building up tactile expectations by rhythmic tactile stimulations. These tactile expectations are every now and then violated by omitting otherwise expected stimuli (Figure 1). The inter-stimulus interval was 3,000 ms. Around every 25 trials, and always starting after an omission, periods of non-stimulation occurred that would last 15 s. The first 6 s worked as a wash-out period, and the remaining 9 s were cut into three epochs of non-stimulation. There are thus nine trigger values in the data responding to nine different kinds of events (Table 1).

**Table 1:** Mapping of trigger values and annotated events.

**Trigger value**	**Annotation**	**Notes**	**Number of trials**
1	*Standard 1*	First stimulation	~200
2	*Standard 2*	Second stimulation	~200
3	*Standard 3*	Third stimulation	~200
4	*Standard 4*	Fourth stimulation	~135
5	*Standard 5*	Fifth stimulation	~66
13	*Omission 4*	Omission following third stimulation	~66
14	*Omission 5*	Omission following fourth stimulation	~66
15	*Omission 6*	Omission following fifth stimulation	~66
21	*Non-Stimulation*	Absence of stimulation outside the rhythmic stimulation sequences	~130

During the stimulation procedure, participants were watching an unrelated nature programme with sound being fed through sound tubes into the ears of participants at ~65 dB, rendering the tactile stimulation completely inaudible. Participants were instructed to pay full attention to the movie and no attention to the stimulation of their finger. In this way, expectations should be mainly stimulus driven, and thus not cognitively driven or attention driven. Information about the labeling of triggers and numbers of trials can be seen in Table 1.

An analysis of induced responses will be carried out. It is known from many experiments that tactile stimulations are followed by a desynchronization in the alpha and beta bands. The desynchronization is followed by the so-called beta rebound, a subsequent increased synchronization (Salmelin and Hari, 1994; Salmelin et al., 1995). Beamformer source reconstructions will be made based on the beta rebound. For both analyses in sensor and source space, a statistical comparison will be made between *Standard 1* and *Standard 3*. We will explore whether the beta rebound differs between novel (*Standard* 1) and repeated (*Standard 3*) stimulations. The specific parameters going into the analysis will become apparent in the analysis steps below.

### Preparation of subjects

In preparation for the MEG-measurement each subject had their head shape digitized using a Polhemus Fastrak. Three fiducial points, the nasion and the left and right pre-auricular points, were digitized along with the positions of four head-position indicator coils (HPI-coils). Furthermore, about 200 extra points, digitizing the head shape of each subject, were acquired.

### Acquisition of data

Data was sampled on an Elekta TRIUX system at a sampling frequency of 1,000 Hz and on-line low-pass and high-pass filtered at 330 and 0.1 Hz, respectively. The data were first MaxFiltered (–v2.2) (Taulu and Simola, 2006), movement corrected and line-band filtered (50 Hz). MaxFiltering was done with setting the coordinate frame to the head coordinates, setting the origin of the head to (0, 0, 40 mm), setting the order of the inside expansion to 8, setting the order of the outside expansion to 3, enabling automatic detection of bad channels and doing a temporal Signal Space Separation (tSSS) with a buffer length of 10 s and a correlation limit of 0.980. Calibration adjustment and cross-talk corrections were based on the most recent calibration adjustment and cross-talk correction performed by the certified Elekta engineers maintaining the system.

## Analysis

The analysis pipeline is built up around five scripts for analyzing the relevant MEG and MRI data and four scripts for plotting what comes out of the analysis steps (Table 2). Run the script *create_MEG_BIDS_data_structure.m* to set up the folder structure that the remaining functions depend on.

**Table 2:** The 10 scripts that cover all relevant steps of the analysis pipeline.

**Script name**	**Purpose**
*create_MEG_BIDS_data_structure.m*	Create all relevant directories where all data and all figures will be saved
*sensor_space_analysis.m*	Go from raw MEG data to a time-frequency representation for each subject
*mr_preprocessing.m*	Go from raw MRI data to a volume conductor and a forward model for each subject
*source_space_analysis.m*	Extract fourier transforms and do beamformer source reconstructions for each subject
*grand_averages.m*	Do grand averages across subjects for both the sensor and source spaces
*statistics.m*	Do statistics on time-frequency representations and beamformer source reconstructions
*plot_sensor_space.m*	Plot all steps in the sensor space analysis
*plot_processed_mr.m*	Plot all steps in the MR processing
*plot_source_space.m*	Plot all steps in the source space analysis
*plot_grand_averages.m*	Plot grand averages in both the sensor and source spaces, with and without statistical masking

**Figure 2:**
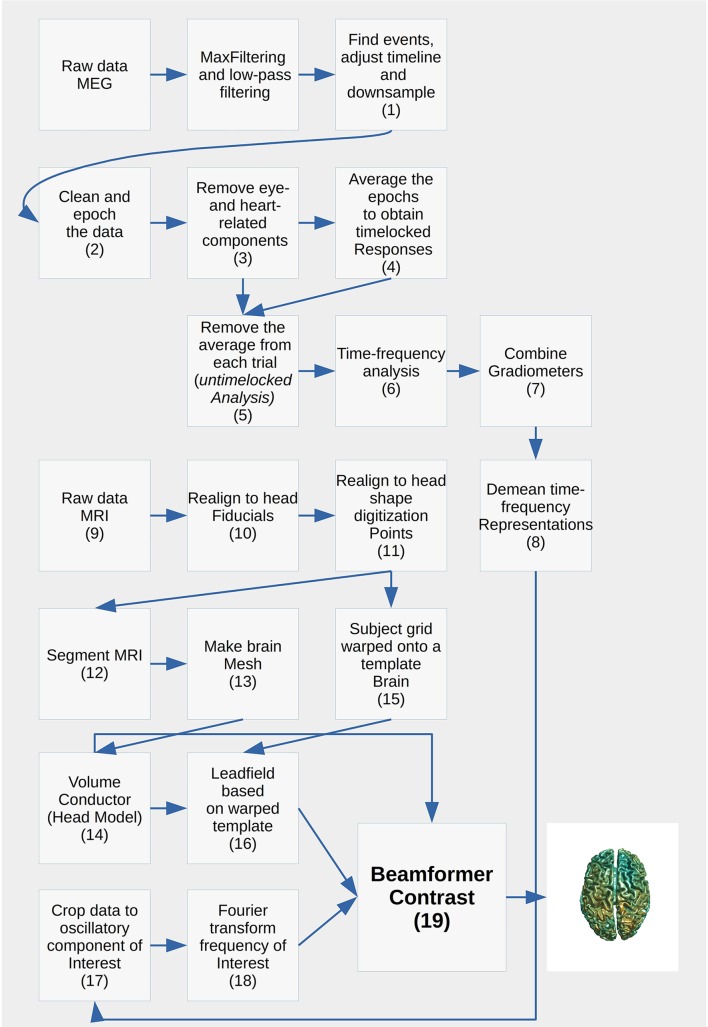
Cookbook for performing a single subject analysis. Numbers point to the sections below.

Each analysis script begins with three sections: SET PATHS, ADD PATHS, and SUBJECTS AND DATES. In the SET PATHS section, *home_dir* should be set to the user's own home directory. ADD PATHS adds FieldTrip and the folders that contain the functions for the analysis scripts (in this example sensor space analysis, Code Snippet 1). SUBJECTS AND DATES contains all the subject names and the dates of their recordings (Code Snippet 1). These three sections are followed by sections that are used to apply the actual analysis to the data. See Figure 2 for an overview of the pipeline for each subject. The boxes on the overview each have a function associated with them which can be accessed from the analysis scripts (Table 2). The analyses have been run with FieldTrip-20170906 (ftp://ftp.fieldtriptoolbox.org/pub/fieldtrip/).

### Goal of analysis

The goal of the analysis is to compare beamformer reconstructed activity between novel and repeated stimulations for the beta rebound statistically. To meet this goal, the following are necessary: (1) induced responses from each subject's raw data are extracted (*sensor_space_analysis.m*, Table 2); (2) Statistics are done on the induced responses for the purpose of identifying when and at what frequency the differences in the beta rebound are statistically significant between novel and repeated stimulations (*statistics.m*, Table 2) (3) volume conductors and forward models are created based on the individuals MRIs (*mr_preprocessing.m*, Table 2); (4) beamformer source reconstructions are made on the individual level (*source_space_analysis.m*, Table 2); (5) statistics are made across the events based on the individual source reconstructions (*statistics.m*, Table 2). Furthermore, scripts are supplied for plotting all steps and calculating grand averages (Table 2). In these analyses, I will focus on the so-called beta rebound (~15–21 Hz) that manifests as an increase in power from around 500 to 1,400 ms after a tactile stimulation (Gaetz and Cheyne, 2006; Gaetz et al., 2010; Cheyne, 2013).

**Code Snippet 1:**
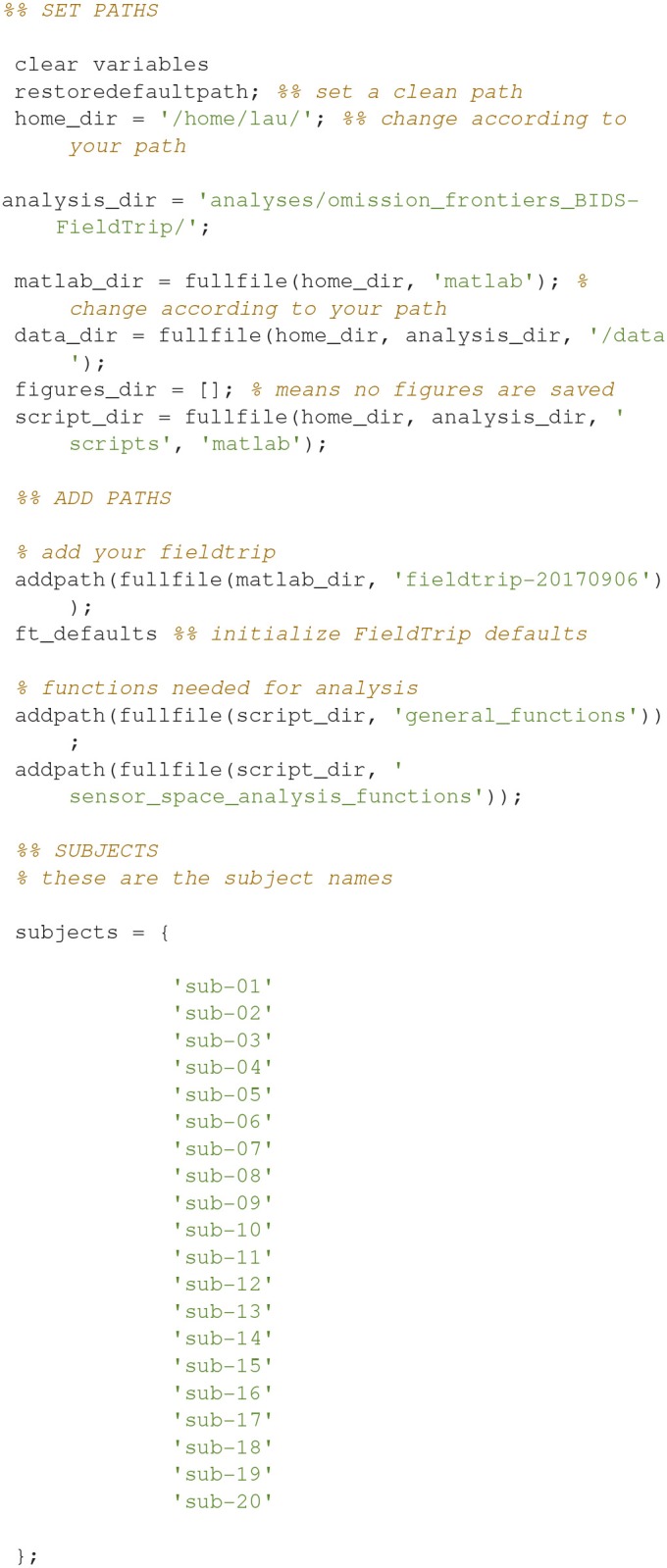
SET PATHS, ADD PATHS, and SUBJECTS AND DATES sections which are used to set up all analysis scripts.

### Understanding the pipeline

The function called *loop_through_subjects.m* (Code Snippet 2) is crucial. This is the function that all pipeline functions below are using. The function is somewhat complicated, but it is very important since it is the one that establishes and maintains the structure and naming of folders and files. The arguments that go into it (Table 3) explicates the idea behind it.

**Table 3:** Arguments for *loop_through_subjects*, which structures input and output of all operations done on single subjects.

**Argument**	**Purpose**
*subjects*	Subject IDs indicating the directory name of the subject
*data_dir*	Whether data is MEG or MRI data
*function_name*	The function that should be applied to all subjects
*cfg*	Configuration structure, as known from FieldTrip
*output*	A cell array of name(s) of the output file(s)
*input*	A cell array of name(s) of the input file(s)
*figures_dir*	Where figures should be stored (leave empty, [], if no figures are produced)
*overwrite*	Whether existing output files should be overwritten

**Code Snippet 2:**
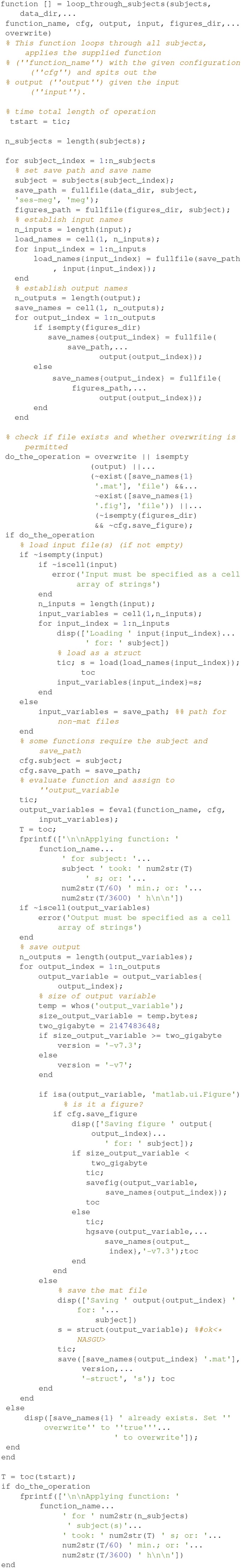
The *loop_through_subjects* function. This function is used to specify input (names), output (names), the function that take the input, the configuration that should be fed to the function. This is applied to all subject recordings in *subjects_and_dates*. Configurations (*cfg*) to FieldTrip functions can be used to easily change how the function is applied.

There is a similar function for doing operations across all subjects at once called *apply_across_subjects.m* (Table 4, Code Snippet not shown here). *loop_through_subjects.m* loops through all subjects, applies a function to all of them with a configuration structure, specifies input and output files and controls whether earlier output should be overwritten. All single subject figures shown below are created from subject *sub-01*. *apply_across_subjects.m* is intended for operations that need to load data from all subjects before the operation can be performed, e.g., grand averages or operations that are applied to grand averages, dependent on the *running_on_grand_average* argument (Table 4). In contrast, *loop_through_subjects* consecutively loops through each subject independently. The application of each of the sub-functions comes with an estimated time for how long it takes to apply, including loading and saving, based on running it on a computer with the following specifications: Memory 126 GiB and 32 processors running at 2.60 GHz.

**Table 4:** Arguments for *apply_across_subjects*, which structures input and output of all operations done across subject.

**Argument**	**Purpose**
*subjects*	Subjects IDs indicating the directory name of the subject
*data_dir*	Whether data is MEG or MRI data
*function_name*	The function that should be applied to all subjects
*cfg*	Configuration structure, as known from FieldTrip
*output*	A cell array of name(s) of the output file(s)
*input*	A cell array of name(s) of the input file(s)
*figures_dir*	Where figures should be stored (leave empty, [], if no figures are produced)
*overwrite*	Whether existing output files should be overwritten
*running_on_grand_average*	Whether the operation should be run on a grand average or whether a grand average should be calculated

## Stepwise procedures

### Sensor space analysis

The sensor space analysis is dependent on the functions in the *sensor_space_analysis_functions* folder. These cover steps from reading in raw data to creating a time-frequency representation (Table 5). All functions have a short documentation about what input they take.

**Table 5:** Functions in the *sensor_space_analysis_functions* folder and a brief description of what their purposes are.

**File names**	**Description**
*trial_function.m*	Describing how trials should be defined (see below)
*define_trials_and_preprocess_data.m*	Defining trials from raw data and subsequently preprocessing it
*clean_data.m*	Exclude high-variance trials using a graphical routine
*run_ica.m*	Decompose the data into independent components
*ica_components.tsv*	Text file for entering components into that should be removed
*remove_components.m*	Remove the components from the text file above
*timelocked_analysis.m*	Finding the average for each of the trial types
*untimelocked_analysis.m*	Removing the average from each trial
*time_frequency_representation.m*	Calculate a time-frequency representation based on the average-cleaned data
*combine_gradiometers.m*	Combine the planar gradiometers into planar gradient magnitudes in the time-frequency representation
*baseline_tfr.m*	Demean the time-frequency representations by subtracting the mean power from the non-stimulation trials

*Functions are put in the order that they are meant to be applied*.

### Trial function

**Figure 3:**
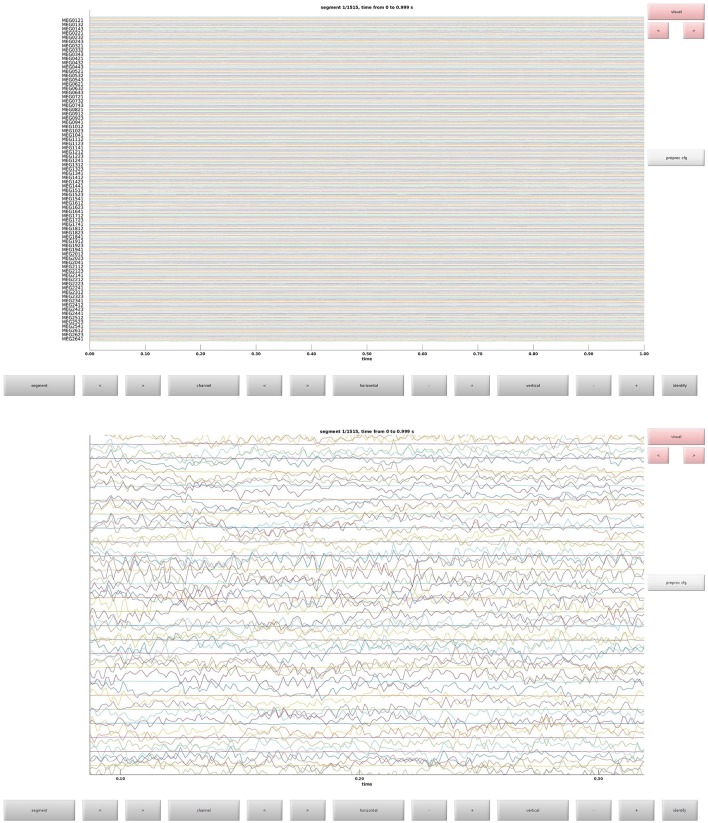
**(Top)** The raw data browser for the example subject. **(Bottom)** A zoom in on some sensors.

This is the function that is used to define trials from the raw data. This defines what parts of the raw data constitute trials and the event codes to be associated with them (Table 1). In Figure 3 the raw data browser can be seen.

### Define trials and preprocess data (1)

Code Snippet 3 shows how the definition of trials from raw data and the preprocessing of data. It also serves as an example of how all analysis steps are carried out for all analysis steps. The second line shows which FieldTrip functions are used (here *ft_definetrial, ft_preprocessing*, etc.). This is always followed by four options that should be set: *overwrite* (should existing output files be overwritten?), *input* [name(s) of input file(s) (.mat format only)], *output* [name(s) of output file(s)] and *function_name* (name of the function that should be applied). Then a configuration (*cfg*) is built and the *loop_through_subjects* function is run to apply the settings to all subjects. The configuration fields *preprocessing* and *trial_definition* are fed directly to *ft_preprocessing* and *ft_definetrial*, respectively.

In the trial definition, the trigger channel, the time in seconds that should be included around the trigger (*pretrigger* and *posttrigger*) and the trial function are entered. In the preprocessing, we only include demeaning based on the duration of the trials. No low-pass filtering is necessary since we are going to do a time-frequency analysis. *adjust_timeline* is used to adjust the offset of the trigger due to a delay between the trigger and the actual stimulation. *downsample_to* is used to reduce sampling rate, and effectively the data size, but it also means that we can only consider frequencies at maximum 100 Hz (Nyquist frequency = half the sampling rate).

Applying the function *define_trials_and_preprocess_data* takes ~5 min per subject.

**Code Snippet 3:**
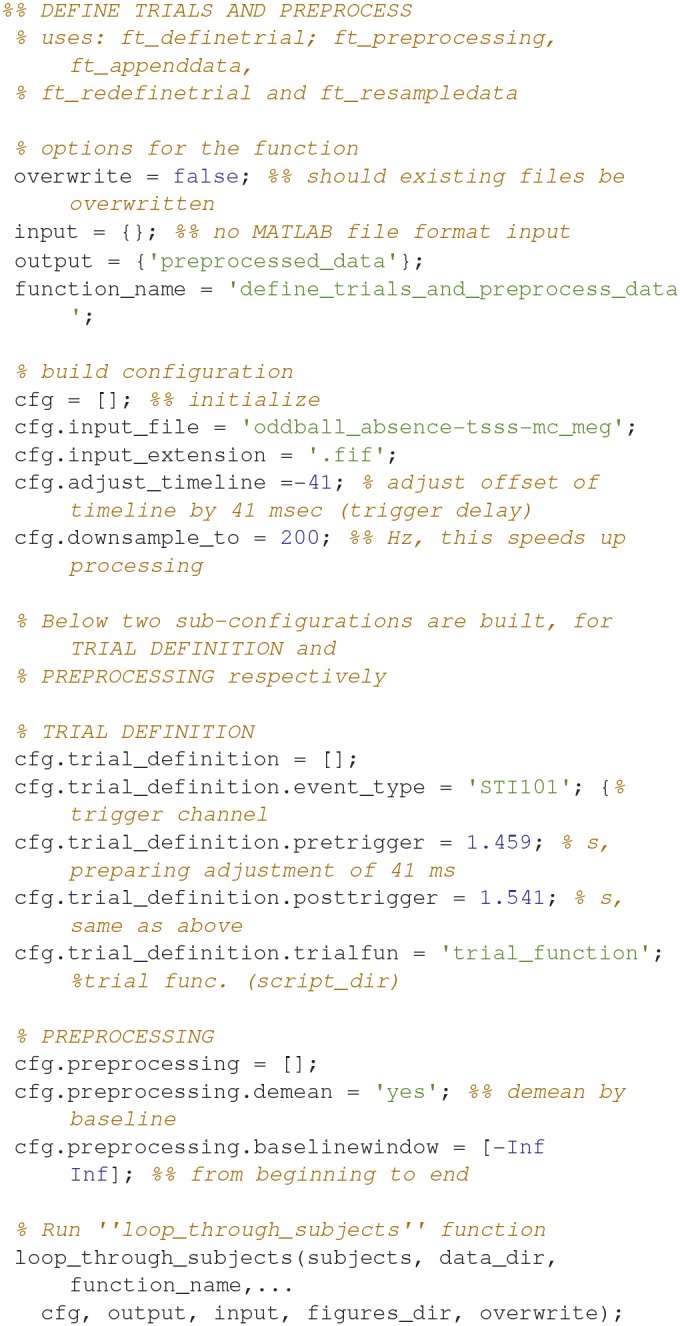
Code for defining trials from raw data and preprocessing data.

### Clean data (2)

**Figure 4:**
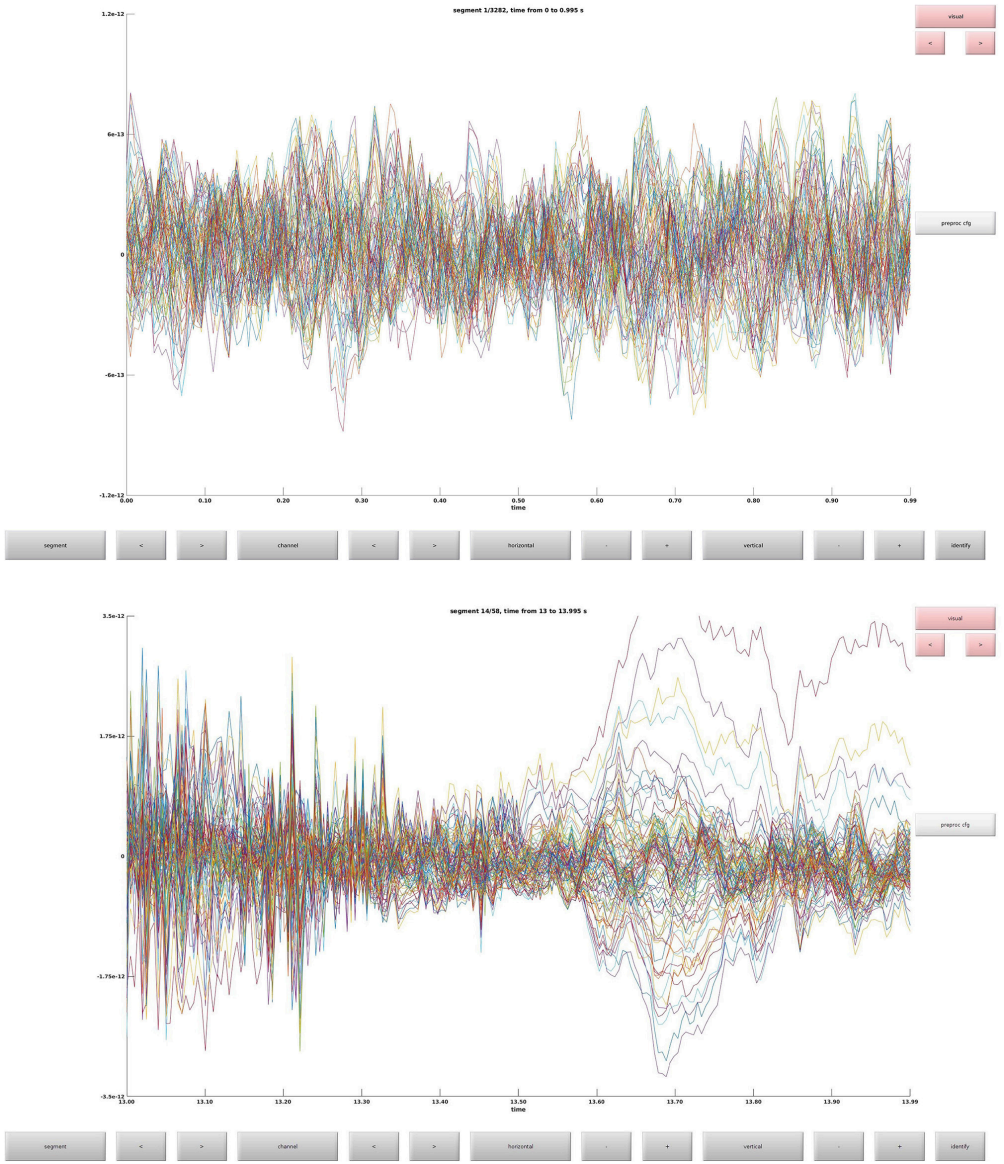
**(Top)** The data browser showing the epoched data. A butterfly plot showing all the magnetometers. Here the first epoch is shown. **(Bottom)** The data browser showing all the magnetometers from one of the removed bad trials.

Clean data sequentially, first magnetometers (MEGMAG) and then gradiometers (MEGGRAD) with graphical aid (Code Snippet 4). High-variance trials should be removed. The indices for the removed trials is written to a tsv-file (tabulator separated values). An example plot of the cleaned epochs can be seen in Figure 4.

How long that the function *clean_data* takes to apply is dependent on user input.

**Code Snippet 4:**
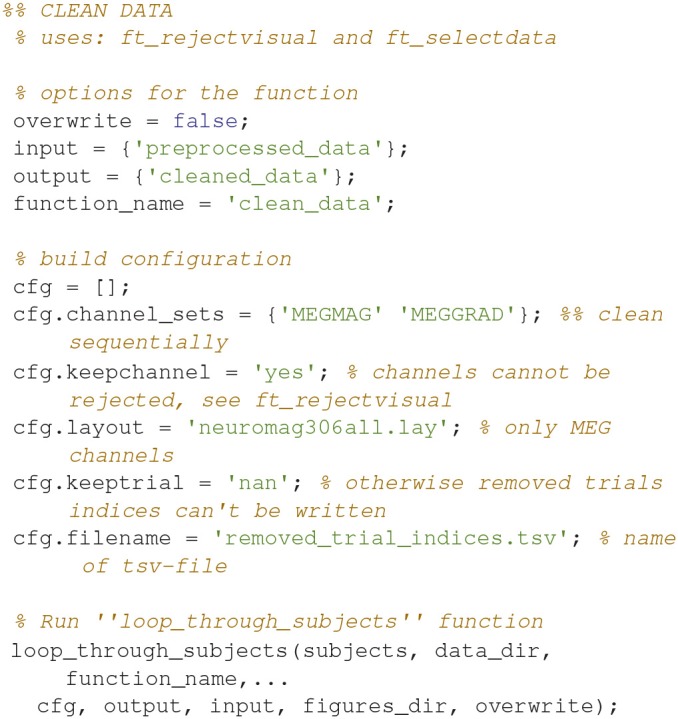
Code for cleaning the preprocessed data.

### Run independent component analysis (3)

**Figure 5:**
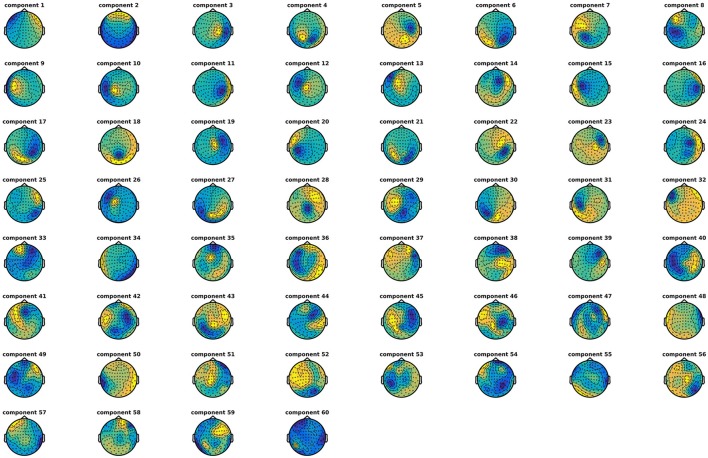
The components found from the independent component analysis.

Decompose data into 60 independent components (Code Snippet 5). In these components, it is often possible to identify components related to eye blinks, eye movements, and heart beats. The resultant components can be seen in Figure 5. The number of components chosen, 60, reduces the dimensionality of the data. After MaxFiltering data dimensionality is reduced from 306 dimensions, corresponding to the number of channels, to a range between 60 and 70 independent dimensions. Reducing the data to 60 independent components is thus not reducing the dimensionality much more than the application of MaxFiltering already did. A particular issue that may arise when using ICA is that some components, say the heart beat component, may not be identifiable in all subjects. This would mean that it would not be possible to process all subjects in the same manner. There may be several reasons for this, e.g., the heart beat signal is only very weakly represented in the MEG data, as may happen for subjects where the distance between the heart and the head is great, i.e., tall subjects, or it may simply be that the recording is too noisy to faithfully record the electrocardiogram. The problem of having differently processed subjects is greatest in between-group studies where having different signal-to-noise ratios between groups may bias results. In within-group studies, the problem is thus less severe, since the decreased signal-to-noise ratio will apply to all conditions the given subject participated in, if ICA is run on all conditions collapsed, as is the case here. Alternative strategies for eye blinks and eye movements is to manually or automatically reject trials that contain eye blinks or excessive eye movements. Following the suggestions for good practice by Gross et al. (2013) one should describe the ICA algorithm (runica: Code Snippet 5), the input data to the algorithm (the epoched data: Code Snippet 5), the number of components estimated (60: Code Snippet 5), the number of components removed (two components: Figure 5) and the criteria for removing them [the likeness to eye blink, eye movements, and heart beat templates (Hyvärinen and Oja, 2000; Ikeda and Toyama, 2000; Jung et al., 2000) and seeing activity in the time courses of the components corresponding to what is recorded with electrooculographic and electrocardiographic channels (can be plotted with *plot_ica* from *plot_sensor_space.m*)]. It should also be mentioned that one can use semi-automatic procedures as to whether components are likely to be related to eye blinks or heart beats (Andersen, this issue).

Applying the function *run_ica* takes ~8 min per subject.

**Code Snippet 5:**
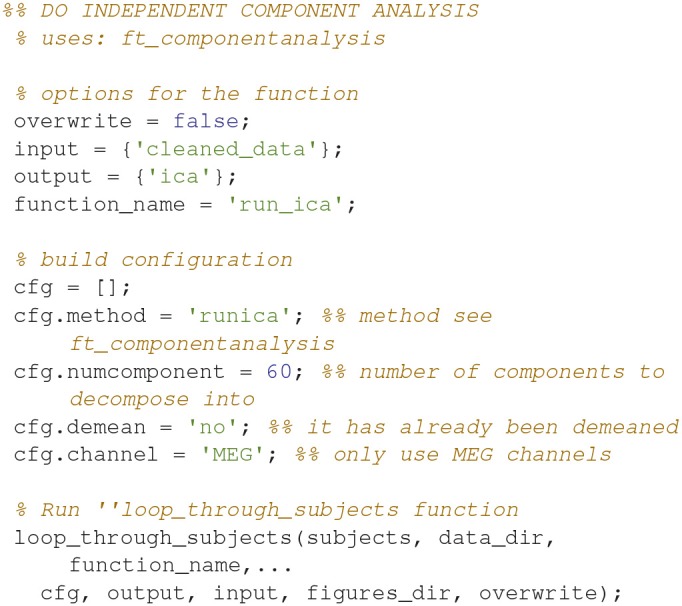
Code for decomposing the data into independent components.

### ICA components (3)

An example of how the components numbers should be entered into the file, *ica_components.tsv*, for each subject can be seen in Table 6. These are also the components that were removed from the present data.

**Table 6:** Components removed for eye blinks, eye movements and heart beats.

**Eye blinks**	**Eye movements**	**Heart beats**
1	2	NaN

*NaN means that a component was not identified*.

### Remove components (3)

Remove the components entered into *ica_components.tsv* from the cleaned data (Code Snippet 6) to remove the orthogonal contributions from eye blinks, eye movements, and heart beats.

Applying the function *remove_components* takes ~2 min per subject.

**Code Snippet 6:**
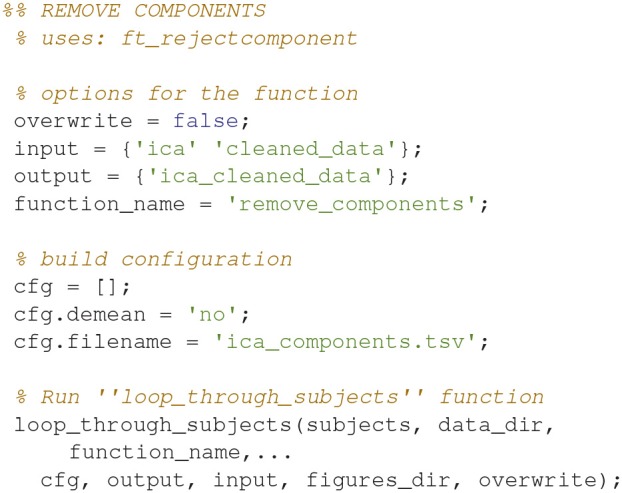
Code for removing the components entered into *ica_components.tsv* from the epoched data.

### Timelocked analysis (4)

**Figure 6:**
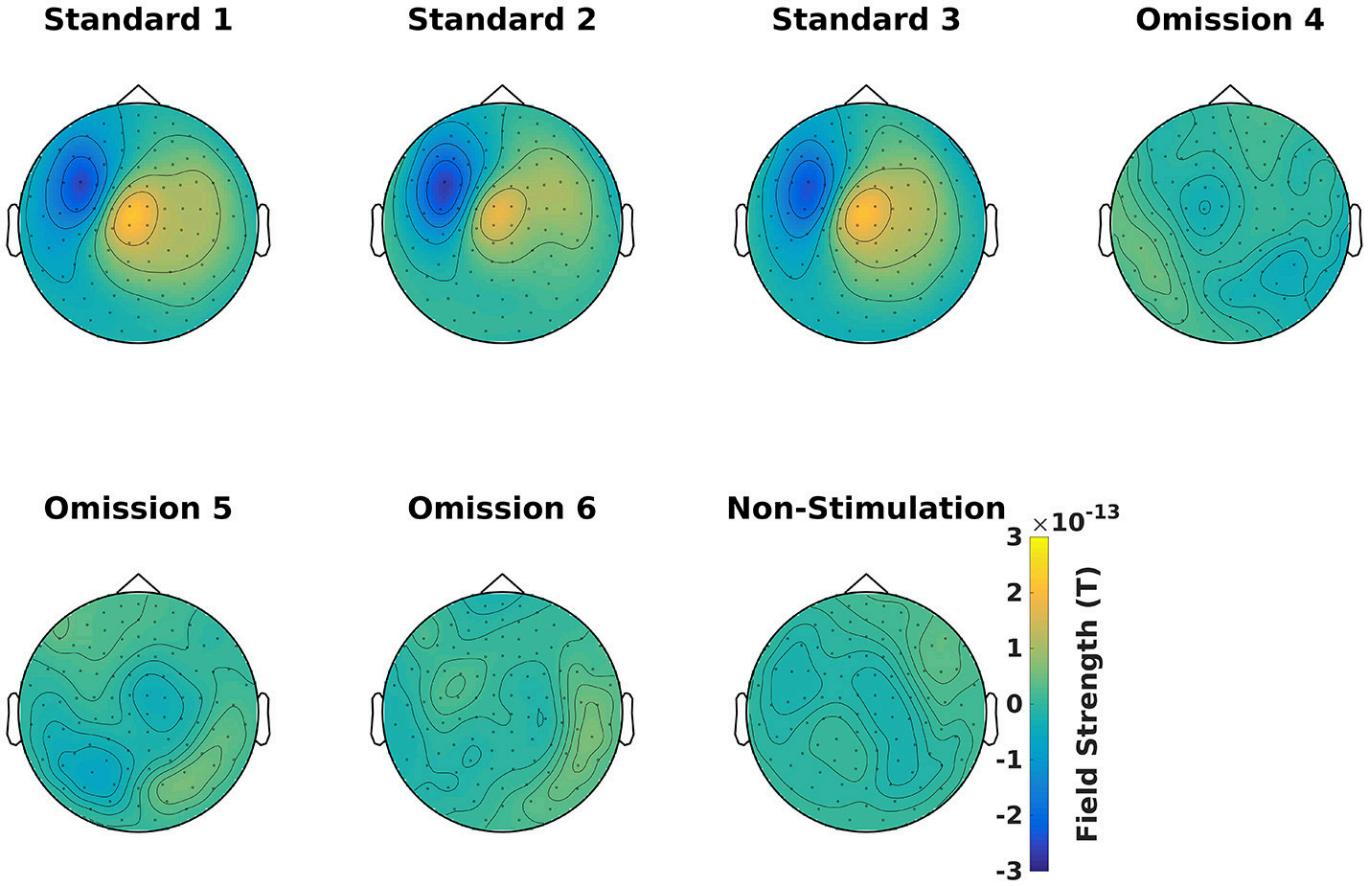
Magnetometer topographical plots for averages from 50 to 70 ms, showing a dipolar pattern typical for activation of the somatosensory cortex. Scale is the same for all plots.

Find the averages for each condition (Code Snippet 7). Example topographical plots can be seen in Figure 6.

Applying the function *timelocked_analysis* takes < ~45 s per subject.

**Code Snippet 7:**
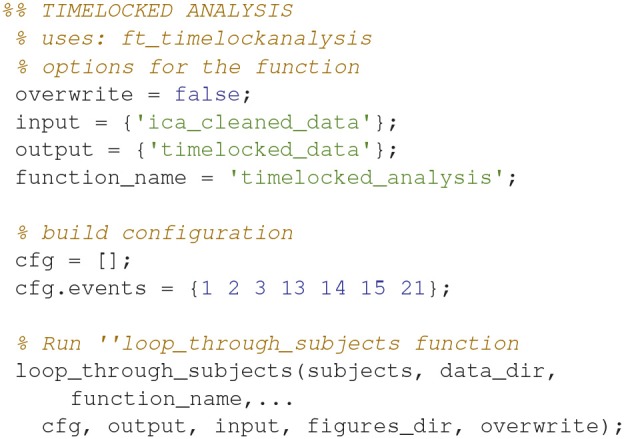
Code for averaging the epochs.

### Untimelocked analysis (5)

Remove the average response from each trial (Code Snippet 8). This is done to minimize how much the timelocked response is present in the subsequent time-frequency representations.

Applying the function *untimelocked_analysis* takes ~1.5 min per subject.

**Code Snippet 8:**
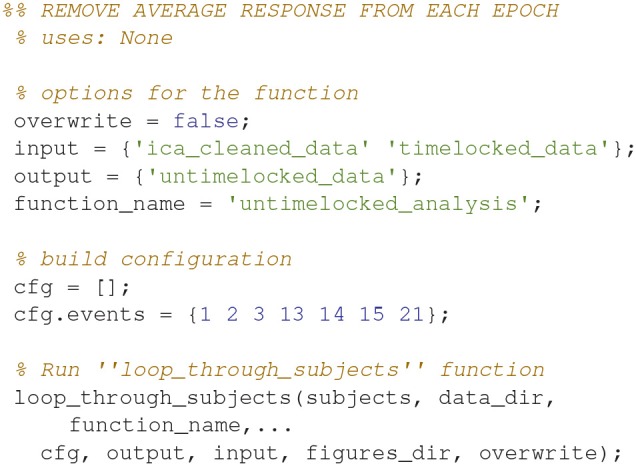
Code for removing the averaged response from each epoch.

### Time-frequency representation (6)

Calculate the time-frequency representations for all of the conditions (Code Snippet 9). This estimates the power in each frequency for each time point based on a wavelet with width 7.

Applying the function *time_frequency_representation* takes ~70 min per subject.

**Code Snippet 9:**
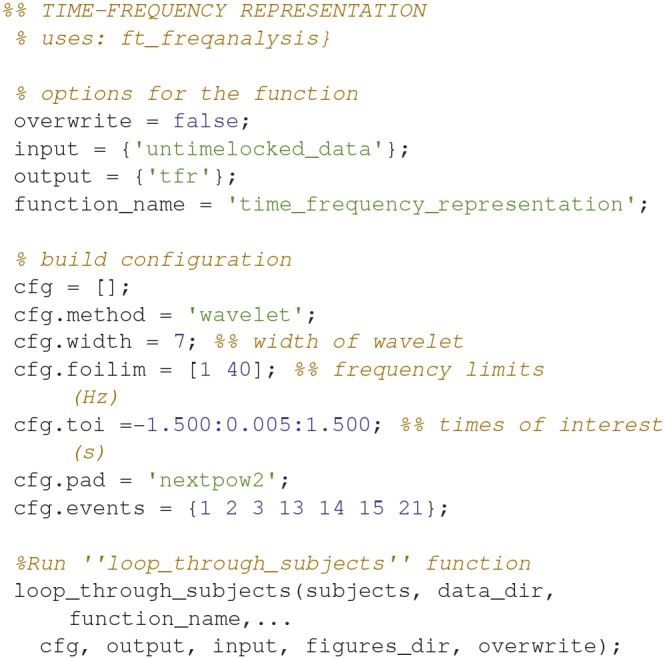
Code for calculating the time-frequency representation for each condition.

### Combine gradiometers (7)

Combine the gradients for each pair of gradiometers for all of the time-frequency representations (Code Snippet 10) into planar gradient magnitudes. The analysis will focus on gradiometers, since magnetometers are normally quite noisy for time-frequency representations.

Applying the function *combine_gradiometers* takes ~2 min per subject.

**Code Snippet 10:**
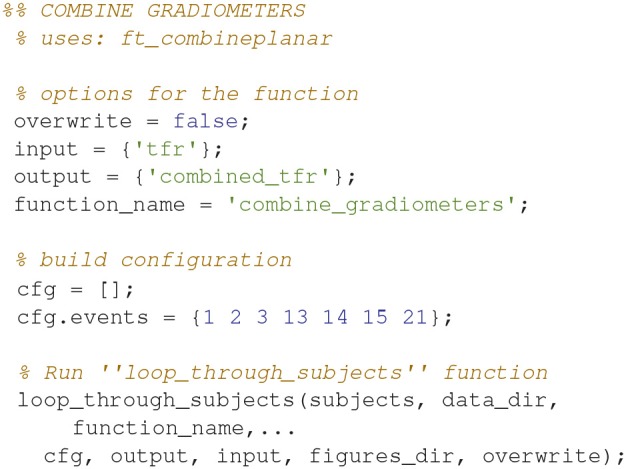
Code for combining the gradiometer data in the time-frequency representation.

### Demean time-frequency representations (8)

**Figure 7:**
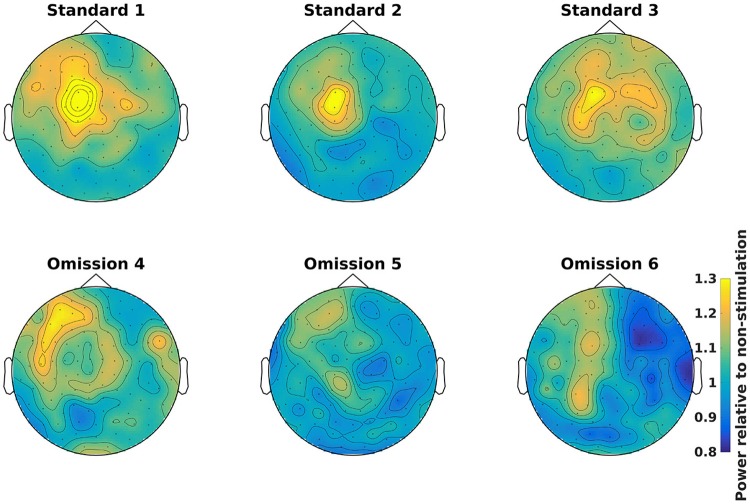
Power topographical plots for Standards and Omissions (baselined with Non-Stimulation) based on gradiometers averaged over 500 to 900 ms and 15 to 21 Hz (the beta rebound). Scale is the same for all plots.

Demean all time-frequency representations with the non-stimulation time-frequency representation (Code Snippet 11). Power relative to non-stimulation can be seen in Figure 7. Absolute power estimates are hard to interpret, and therefore demeaning by a common condition, non-stimulation, makes the time-frequency representations comparable and thus interpretable.

Applying the function *baseline_tfr* takes ~1 min per subject.

**Code Snippet 11:**
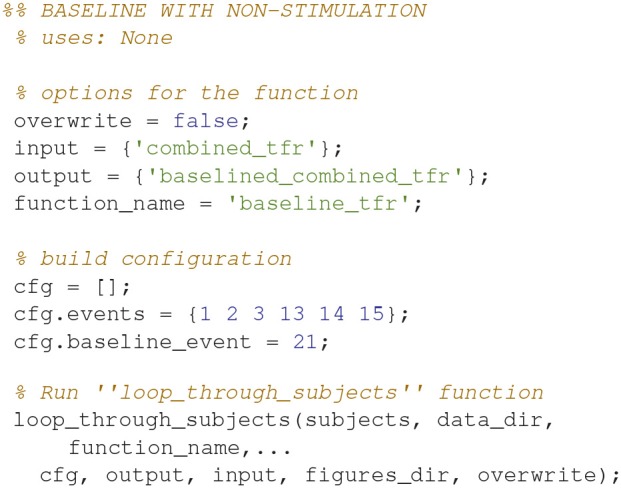
Code for demeaning the time-frequency representation with the non-stimulation time-frequency representation.

## Creating and saving figures

Figures can also be created and saved for each subject by using the *loop_through_subjects* function. As an example, code (Code Snippet 12) is supplied for plotting Figure 7. Scripts for plotting the plots in the manuscript, and several other plots, are all included in the files provided alongside this protocol paper, i.e., *plot_sensor_space.m, plot_processed_mr, plot_source_space*, and *plot_grand_averages*. The user can easily extend the number of plotting functions by modeling them based on the example below (Code Snippet 12). All plotting functions also require a field, *save_figure*, in the configuration (*cfg*). This is a Boolean indicating whether or not the figure should be saved.

**Code Snippet 12:**
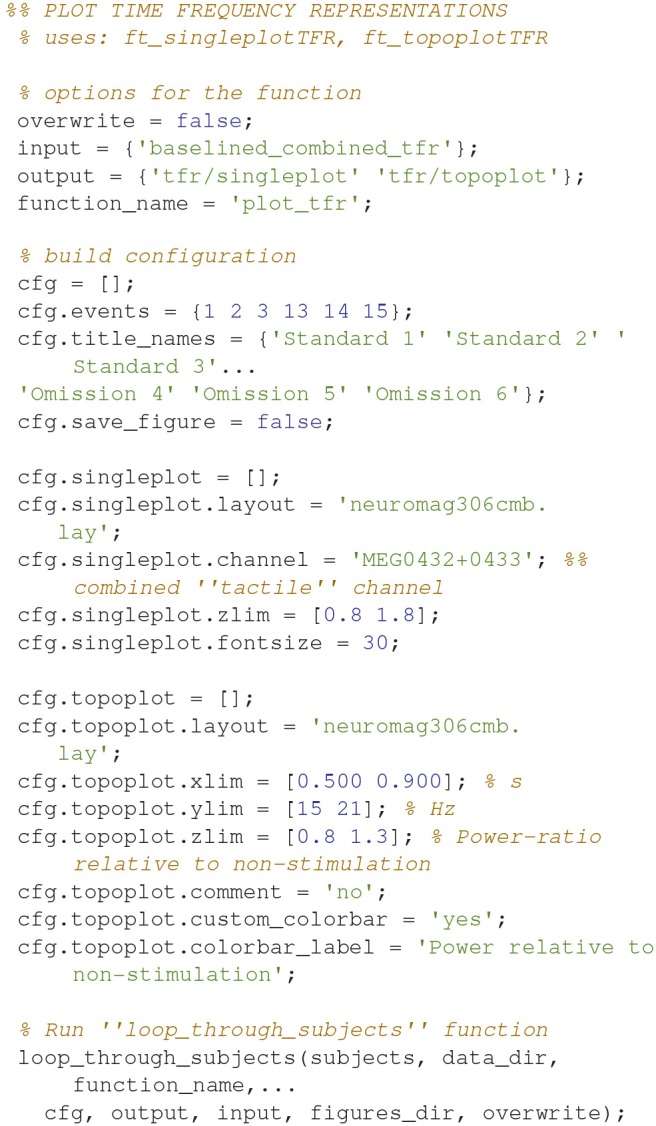
Example code for creating plots of single sensors (not shown here) and topographies (Figure 7) for time-frequency representations. Creating and saving plots for each subject is also done with *loop_through_subjects*.

## Mr-preprocessing

The preprocessing of MR-data is dependent on the functions in the *mr_preprocessing_functions* folder. The names of these functions and a short description of their applications can be seen in Table 7. These cover all steps from reading in the MR-data, through realigning and segmenting, and finally creating a head model (volume conductor) and a leadfield (forward model) for each subject. Due to reasons of anonymity, the downloadable data will not contain the raw MRI data, such that the first three functions cannot be applied to the downloadable data (Code Snippets 13–16). The functions are included though, so that the user can apply to data of his own. The output of *segment_mri.m* is included in the downloadable data, so the analysis can be started from there.

**Table 7:** Functions in the *mr_preprocessing_functions* folder and a brief description of what their purposes are.

**File names**	**Description**
*read_dicoms.m*	Read in an MRI based on the dicoms
*realign_to_fiducials.m*	Realign the MRI to the fiducials
*realign_to_digitization_points.m*	Realign the MRI to the head shape digitization points
*segment_mri.m*	Segment the MRI into the brain, skull and scalp
*make_brain_mesh.m*	Make a mesh based on the segmented brain
*make_headmodel.m*	Make a head model (volume conductor) based on the mesh
*make_warped_grid.m*	Make a subject-grid warped onto a template brain
*make_warped_leadfield.m*	Make the lead field (forward solution) based on the warped grid

*Functions are put in the order that they are meant to be applied*.

### Read dicoms (9)

Create an MRI MATLAB structure based on reading in the dicoms with *ft_read_mri* (Code Snippet 13).

**Code Snippet 13:**
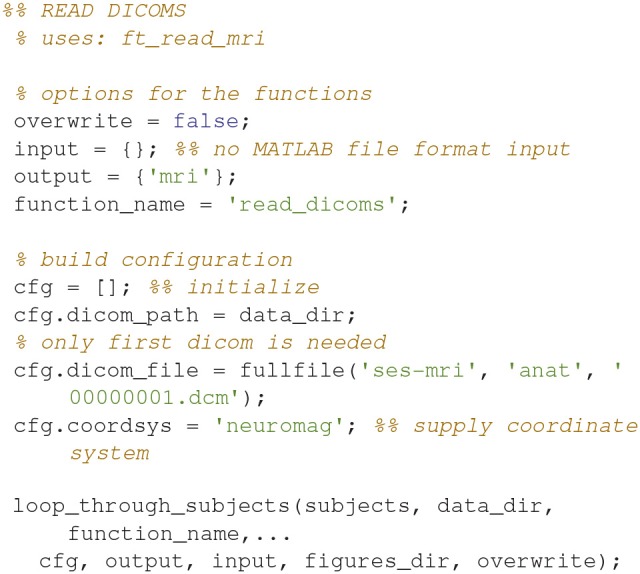
Code for creating an MRI-structure based on reading in the dicoms.

### Realign to fiducials (10)

Align the MR-image to the fiducials (Code Snippet 14). This is done to make the first alignment to the head shape of the subject that was digitized with a Polhemus Fastrak. The fiducials that the MRI should be aligned to are the nasion and the left and right pre-auricular points, but these may differ depending on the acquisition device used.

**Code Snippet 14:**
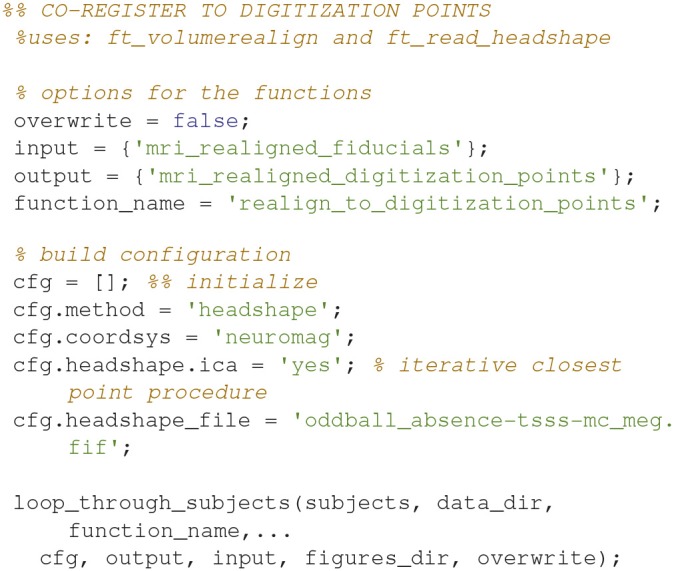
Code for opening the interactive alignment tool for aligning MRI with fiducials.

### Realign to digitization points (11)

Align the fiducial-aligned MRI to of the head shape digitization points digitized with the Polhemus Fastrak (Code Snippet 15). This is done to further optimize the alignment between the head of the subject and the MR-image recorded. The code below relies on an interactive alignment procedure where the user can displace, rotate and scale the head such that they align with the digitization points. The recommended procedure is to make a rough alignment such that the nose from the head model and the outline of the nose digitized with the Polhemus Fastrak roughly align. Subsequently the iterative closest point procedure (*cfg.headshape.ica* Code Snippet 15) is used to minimize the distance between the head shape based on the MRI and the head shape based on the digitization points. This realignment should always be checked, which can for example be done by running *ft_volumerealign* again.

**Code Snippet 15:**
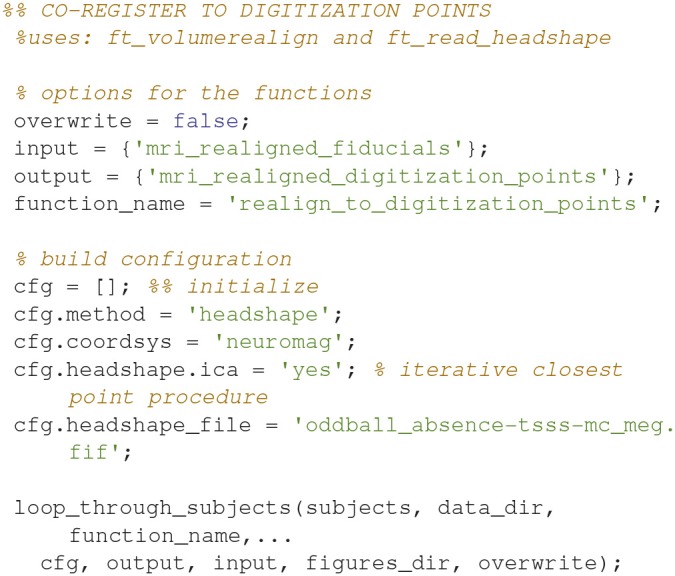
Code for opening the interactive alignment tool for further aligning the fiducial-aligned MRI with the extra head shape digitization points acquired with the Polhemus Fastrak.

### Segment the MRI (12)

Segment the MR-image into brain, skull and scalp using *ft_volumesegment* (Code Snippet 16). This is necessary since sources giving rise to MEG activity are assumed to only exist in the brain.

**Code Snippet 16:**
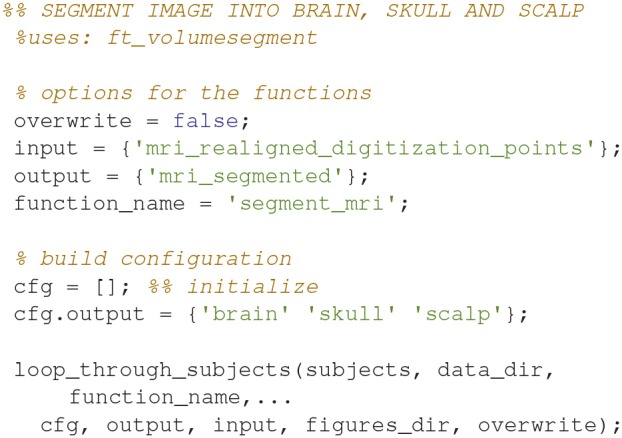
Code for segmenting the brain into the three tissue types: brain, skull and scalp.

### Make a brain mesh (13)

**Figure 8:**
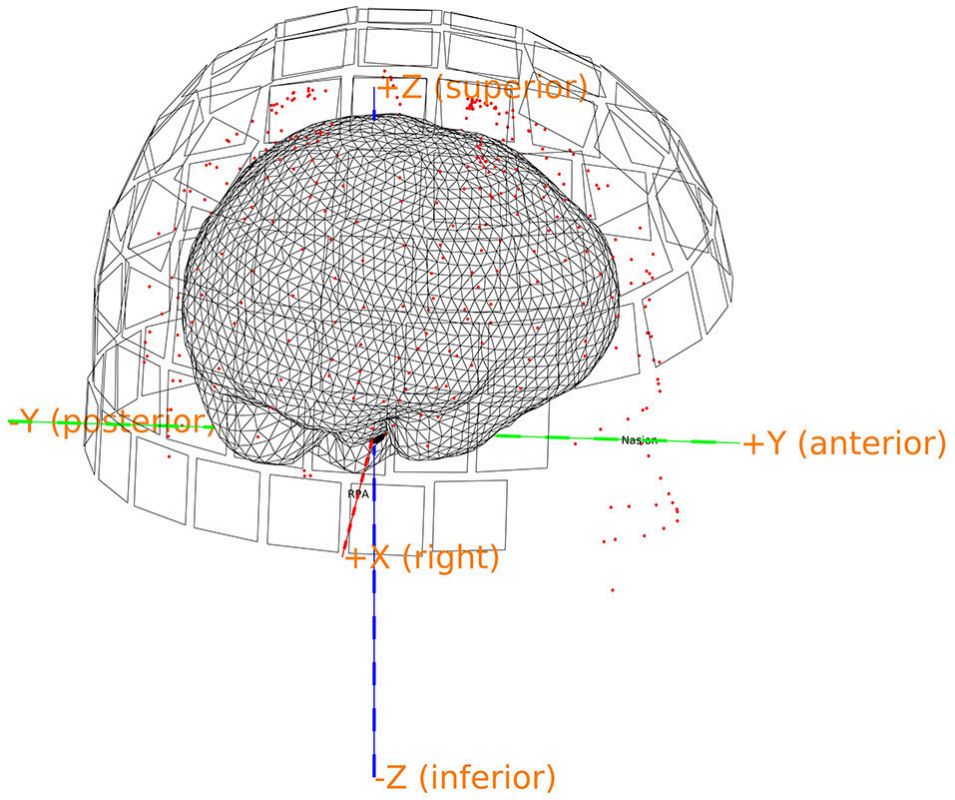
Quality control figure showing the brain, the digitization points, the sensors and the axes. This figure indicates if the realignment process has gone well. More quality figure checks are included in the pipeline.

Make a brain mesh out of the segmented MRI with *ft_prepare_mesh* (Code Snippet 17). At this point a number of quality control figures can be made using *plot_source_space.m* (for an example, see Figure 8). The mesh is a triangulation of the brain based on 3,000 vertices.

Applying the function *make_brain_mesh* takes ~5 s per subject.

**Code Snippet 17:**
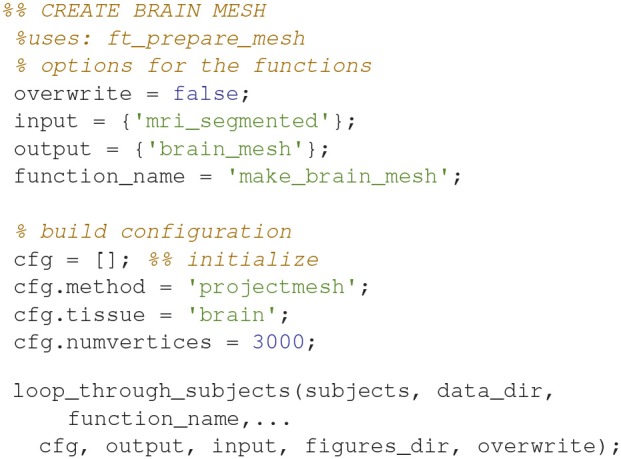
Code for preparing a brain mesh out of the segmented MRI.

### Make a head model (14)

Make a head model (volume conductor) out of the prepared mesh with *ft_prepare_headmodel* (Code Snippet 18). A head model is a volume that specifies how the magnetic fields are conducted through the brain.

Applying the function *make_headmodel* takes ~1 s per subject.

**Code Snippet 18:**
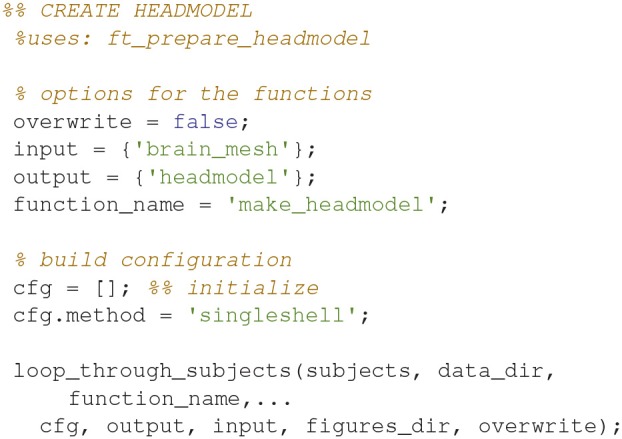
Code for making a head model (volume conductor) out of the prepared brain mesh.

### Make a subject-grid warped onto a template brain (15)

Make a grid where the subject's MRI is warped onto a template brain with *ft_prepare_sourcemodel* (Code Snippet 19). The points on this grid that are inside the brain are the modeled sources of the source model. The warping means that the source reconstructions based on these source models can be compared across subjects.

Applying the function *make_warped_grid* takes ~1 min per subject.

**Code Snippet 19:**
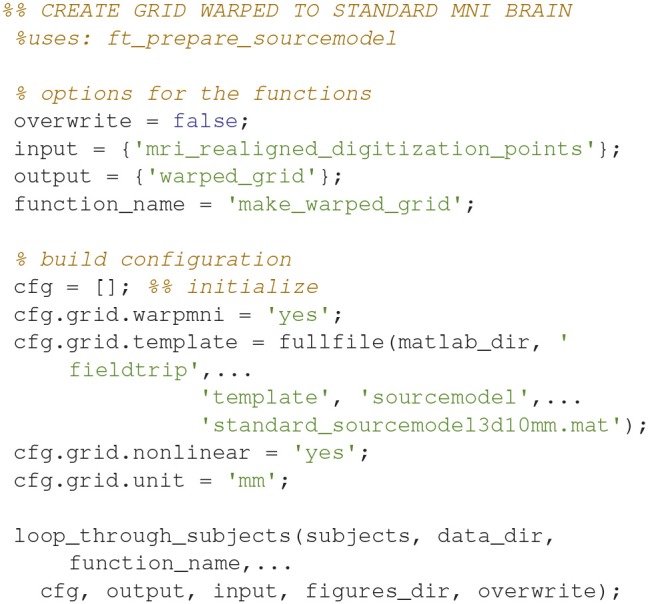
Code for making a grid where the subject's MRI is warped onto a template brain.

### Make the lead field based on the warped grid (16)

**Figure 9:**
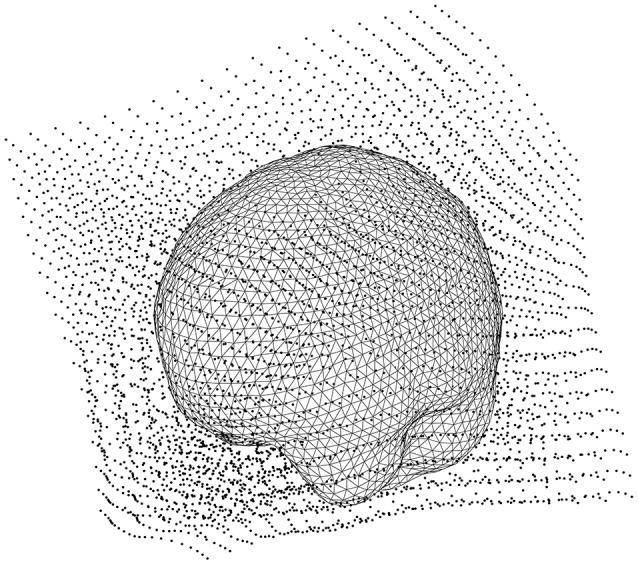
The head model (volume conductor) inside the grid that has been warped to a common template.

Make the lead field based on the warped grid with *ft_prepare_leadfield* (Code Snippet 20). The brain mesh in the warped grid can be seen in Figure 9. The lead field models how the sensors will detect sources from any sources on the grid (inside the brain).

Applying the function *make_leadfield* takes ~3 min per subject.

**Code Snippet 20:**
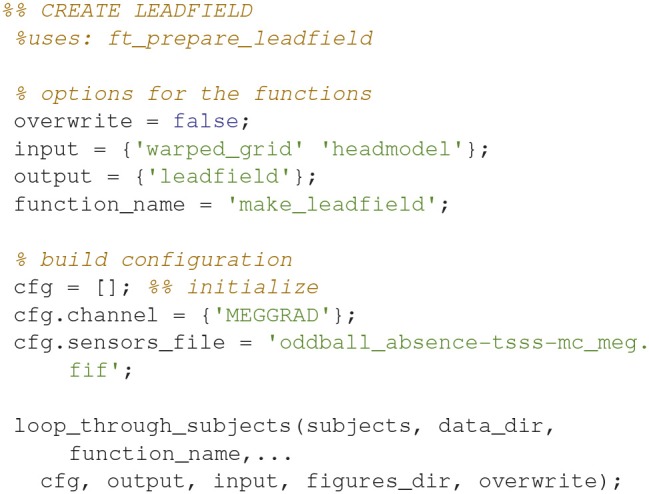
Code for calculating the lead field (forward model) for all the sources of the warped grid that are contained by the brain.

## Statistics—sensor space

The strategy used here will be to do statistics in the sensor space (Table 8) to find the time period in the beta rebound (~15–21 Hz) where the differences between novel (*Standard 1*) and repeated *(Standard 3*) stimulations are the greatest. Subsequently, the beamformer will be done on this time-frequency range. This strategy is one that one should be careful with since it may result in double dipping if anything that is found to be significant is reconstructed. In this example we have mitigated the risk of double dipping, since we specified we would test the beta rebound giving an approximate time range (500–1,400 ms) and frequency range (15–21 Hz), but we did not specify the exact time range and the exact frequency we would reconstruct for the purposes of comparing novel and repeated stimulations. In an ideal hypothesis testing study, both the time range and the frequency range would have been specified exactly beforehand.

**Table 8:** The function related to sensor space operations in the *statistics_functions* folder and a brief description of its purpose.

**File names**	**Description**
*statistics_tfr.m*	Do statistics on the time-frequency representations

### Statistics, time-frequency representation

**Figure 10:**
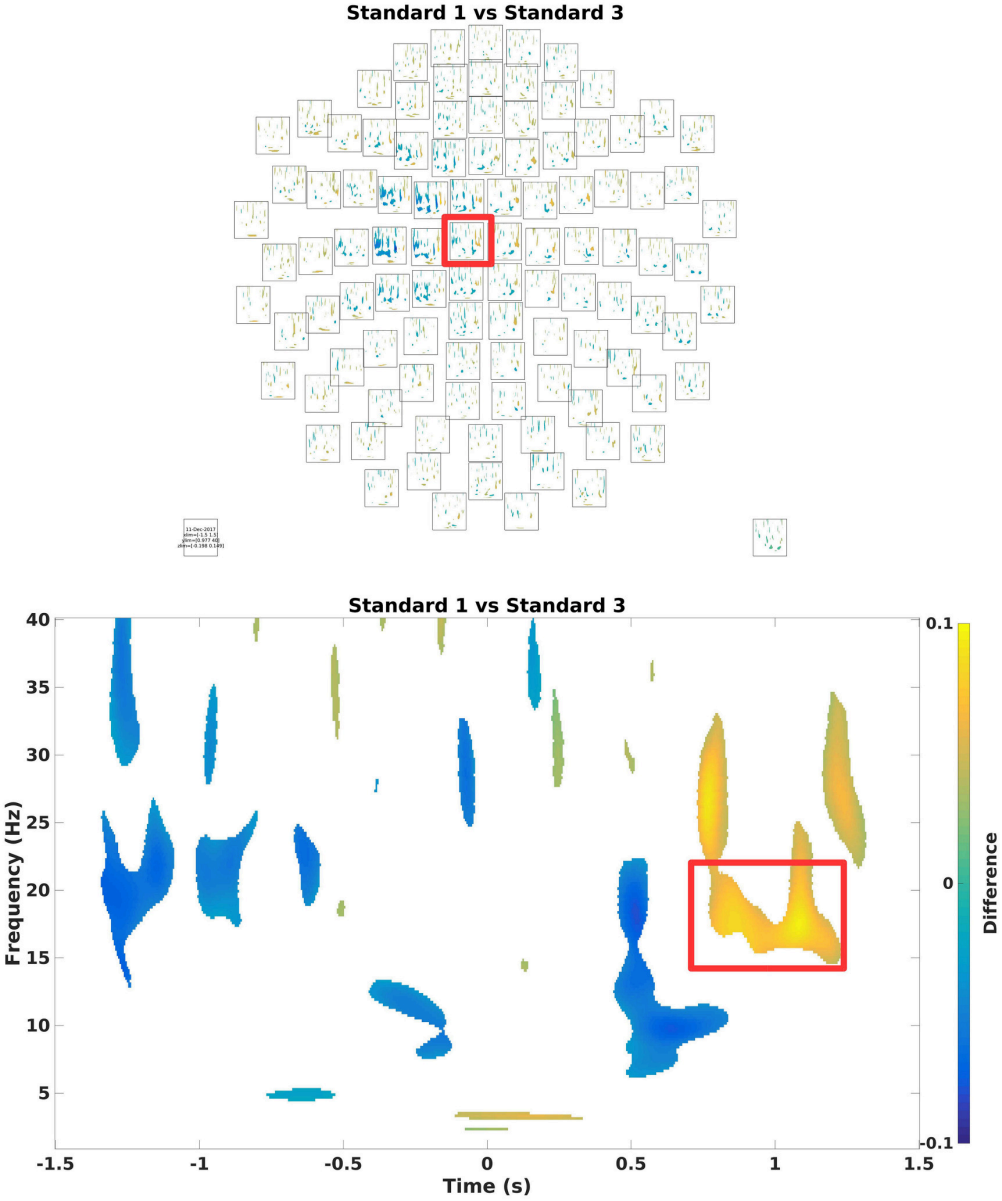
**(Top)** Grand average multiplot masking the non-significant parts. Color shows where there is more/less power for Standard 1 when compared to Standard 3. Red square indicates the sensor shown below. **(Bottom)** Difference in the beta rebound. This is chosen for the subsequent beamformer analysis.

To assess which differences in power arise due to differences in signal and which to change, one can run statistical tests on it (Code Snippet 21). Here, a simple mass-univariate test is run without correction. In Figure 10, a sensor plot can be seen where the non-significant changes (*t*-values < ~-2.09 or *t*-values > ~2.09) have been masked.

Applying the function *statistics_tfrs* takes ~10 min.

**Code Snippet 21:**
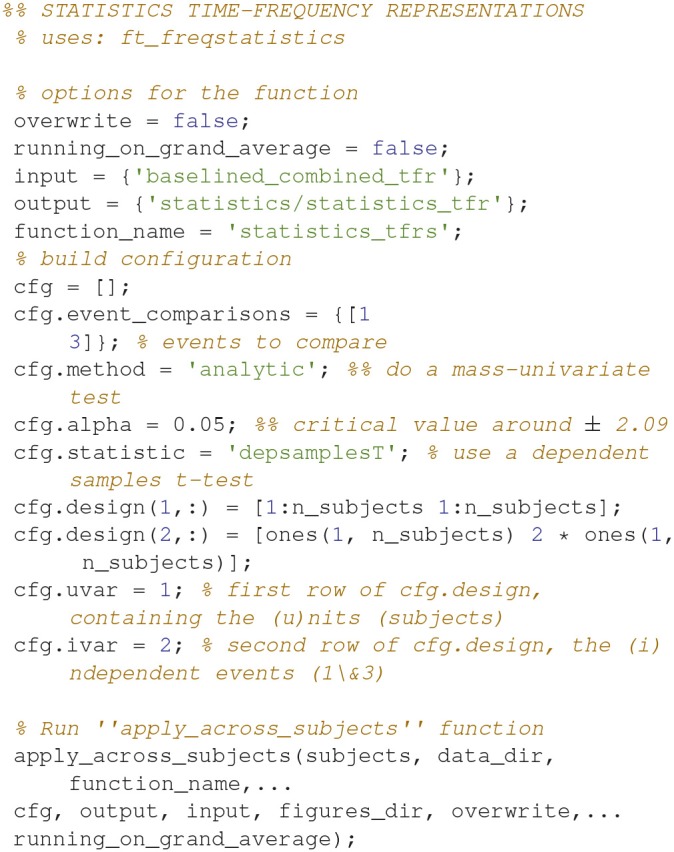
Code for calculating the statistics for the time-frequency representations.

## Source space analysis

The source space analysis is dependent on the functions in the *source_space_analysis_functions* folder (Table 9). First, the untimelocked data are cropped to the time period showing the difference in the beta rebound. Secondly, Fourier transformation is done to estimate the power in the beta rebound frequency range. Finally, beamformer contrasts are estimated based on a contrast against source activity in the non-stimulation trials (Table 1). Optionally, the individual beamformer contrasts can be interpolated onto a common template for visualization if wished for.

**Table 9:** Functions in the *sensor_space_analysis_functions* folder and a brief description of what their purposes are.

**File names**	**Description**
*crop_data.m*	Crop data to the time window of interest
*get_fourier_transforms.m*	Get the Fourier transforms of the frequency of interest
*get_beamformer_contrasts.m*	Get the beamformer localizations for all of the conditions contrasted again the beamformer localization for the non-stimulation condition
*interpolate_beamformer.m*	Interpolate the beamformer localizations onto a common template (only for visualization)

*Functions are put in the order that they are meant to be applied*.

### Crop data (17)

**Figure 11:**
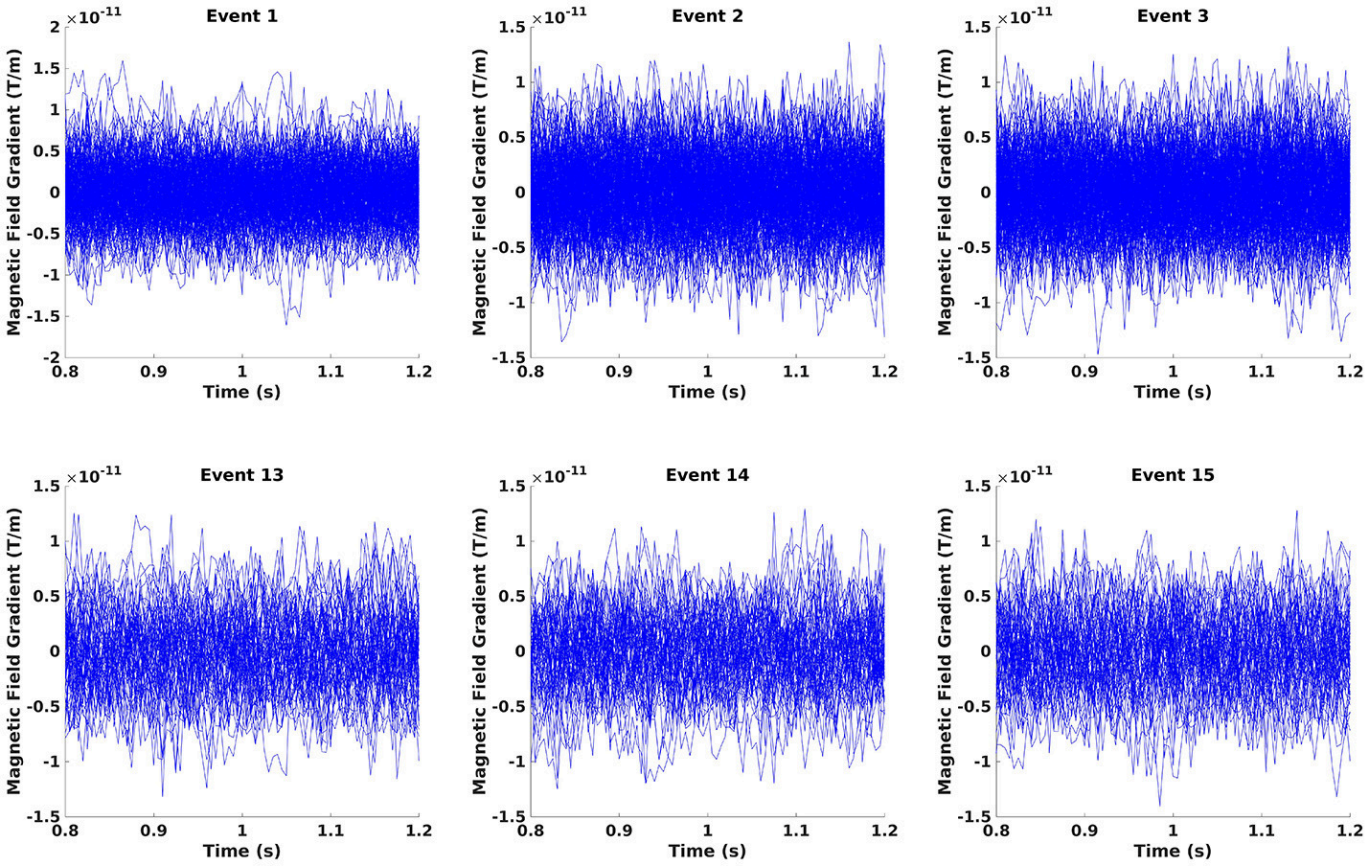
The epochs in the beta rebound where they differ between novel and repeated stimulation (800–1,200 ms). It can be seen that there is no clear timelocked activity.

Crop the data to the time window of interest (Figure 10; Code Snippet 22). The cropped data can be seen in Figure 11. It should be visible that there is no timelocked activity here.

Applying the function *crop_data* takes ~30 s per subject.

**Code Snippet 22:**
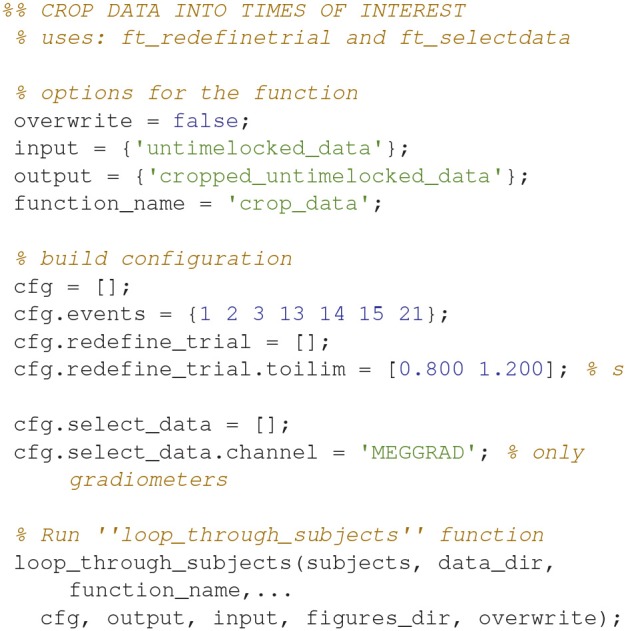
Code for cropping the epoched data into the time window of interest.

### Fourier transforms (18)

**Figure 12:**
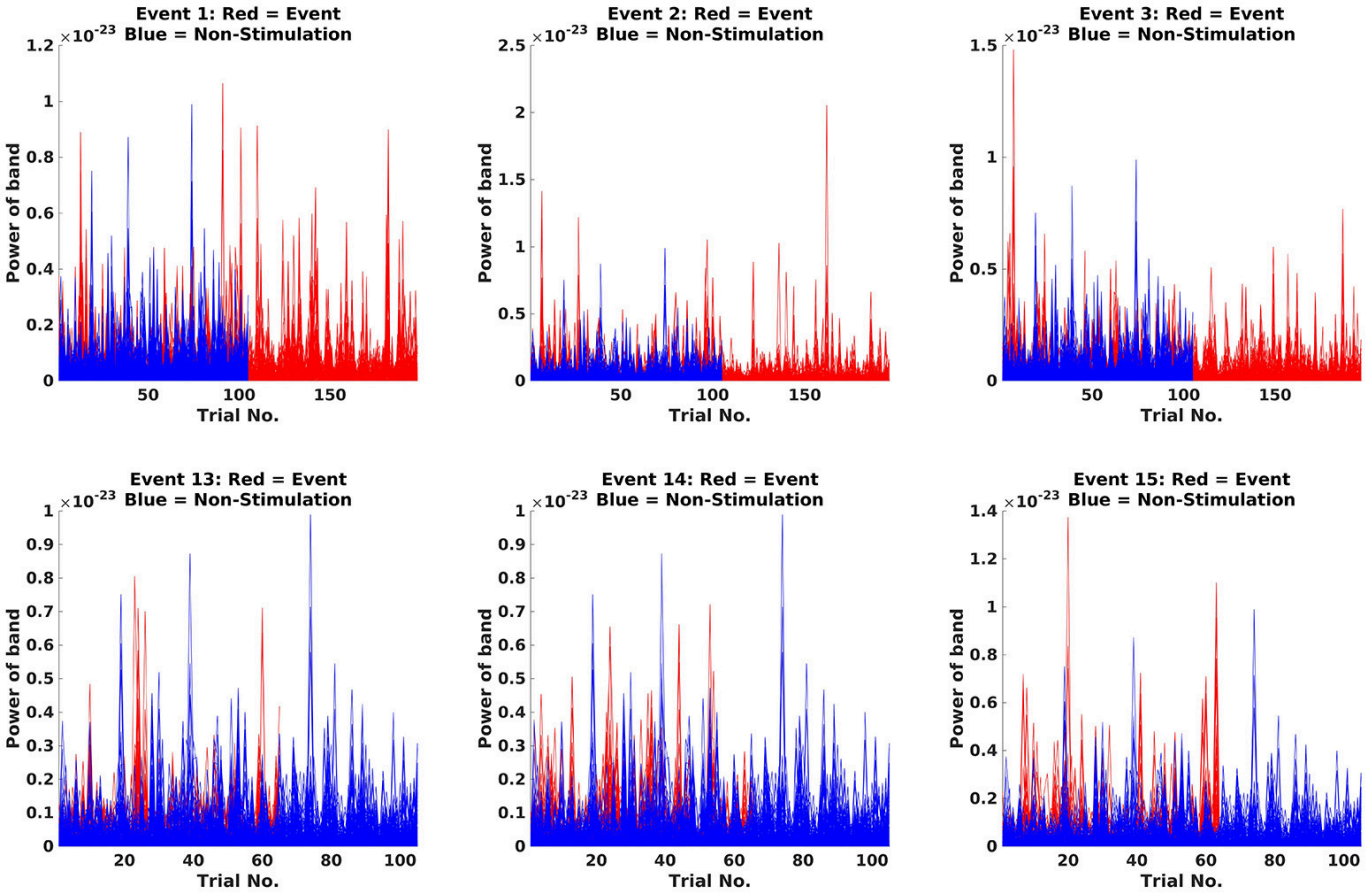
Fourier transforms. On the *y*-axis, power is illustrated, and the *x*-axis shows the trials. For the Standards (red), it can be seen that the power is greater than for Non-Stimulations (blue).

Next step is to make Fourier transforms of the cropped data, focussing on the 18 Hz response (the beta rebound; Code Snippet 23). Estimated power for individual trials can be seen in Figure 12. It can be seen that power in general is higher for stimulations than non-stimulations. Three different transforms are made: one for each of the experimental conditions (*Standards* and *Omissions*), one for the *Non-Stimulations* and one for each of the combinations of each of the experimental conditions and the *Non-Stimulations*. Thus, 13 Fourier transforms are run for each subject.

Applying the function *get_fourier_transforms* takes ~20 s per subject.

**Code Snippet 23:**
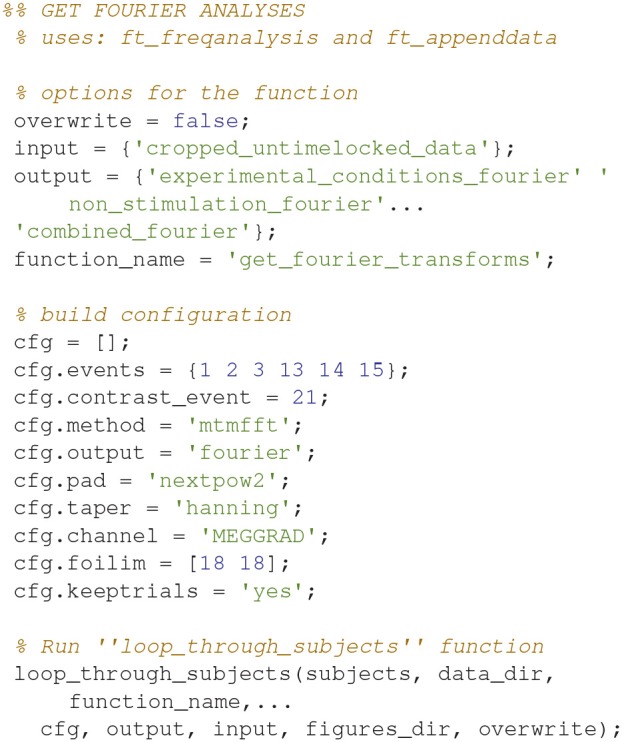
Code for calculating the fourier transforms.

### Beamforming (19)

The actual source reconstruction is done using the non-stimulation trials (Table 1) as a contrast (Code Snippet 24). Beamforming measures the power at each single source point in the brain by applying a spatial filter to each source point to minimize the contribution from all other sources (Gross et al., 2001). The beamforming function (Code Snippet 24) is running three separate beamformers for each experimental condition (*Standards* and *Omissions*). First step is to run a beamformer on the Fourier transform based on the combination between the given experimental condition and the *Non-Stimulation* trials. The spatial filter estimated from the beamforming of that combination is then used for the subsequent beamforming of, second step, the given experimental conditions and, third step, the *Non-Stimulation* trials. Using a common filter makes the two beamforming results comparable. Finally, the beamformer contrast, i.e., between the beamforming of the given experimental condition and the beamforming of the *Non-Stimulation* trials is returned. For a given experimental condition, this reflects where sources are localized to that have greater or lesser power than the *Non-Stimulation* trials do.

Applying the function *get_beamformer_contrasts* takes ~1.5 h per subject if all events are reconstructed.

**Code Snippet 24:**
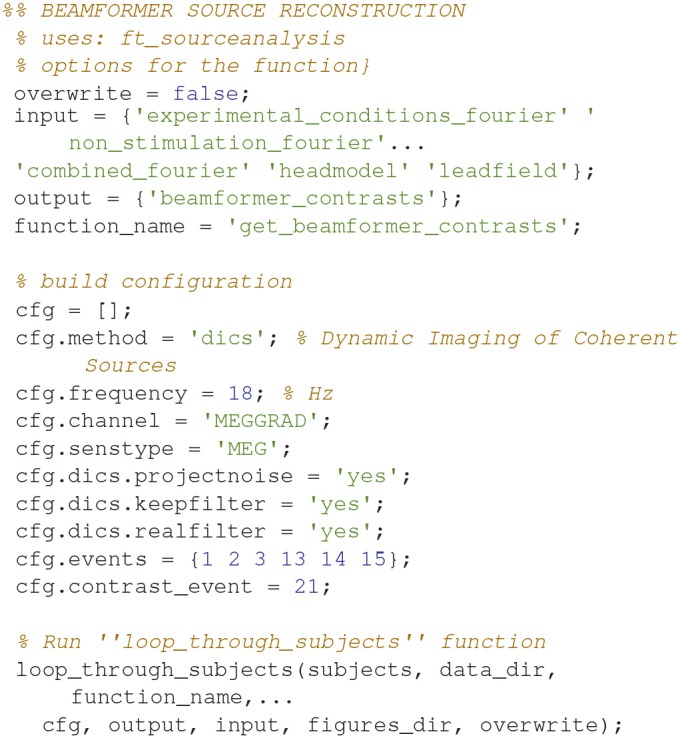
Code for calculating the beamformer solutions based on the Fourier transforms and contrasting them against the non-stimulation cross-spectral density.

## Grand averages

The *grand_averages* script is dependent on the functions in the *grand_averages_functions* folder (Table 10). Note that there is one further option variable, *running_on_grand_average*. This is fed to the new convenience function *apply_across_subjects*, which is very similar to *loop_through_subjects* in its structure, but, as the name implies, *apply_across_subjects*, work on all subjects at the same time. *running_on_grand_average* is simply a logical variable telling *apply_across_subjects* whether subject data for each individual subjects needs to be loaded for the function applied. The grand averages are mostly for visualization.

**Table 10:** Functions in the *grand_averages_functions* folder and a brief description of what their purposes are.

**File Names**	**Description**
*calculate_grand_average_tfr.m*	Get the grand averages for the time-frequency representations
*calculate_grand_average_beamformer.m*	Get the grand averages for the beamformer source reconstructions
*interpolate_grand_average_beamformer.m*	Interpolate the grand for the beamformer source reconstructions onto a common template

*Functions are put in the order that they are meant to be applied*.

### Grand averages, time-frequency representations

**Figure 13:**
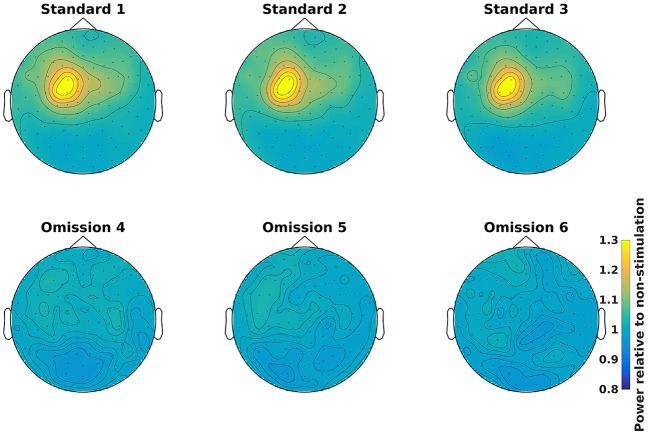
Grand average power topographical plots for Standards and Omissions (baselined with Non-Stimulation) based on gradiometers averaged over 500 to 1,400 ms and 15 to 21 Hz (the beta rebound). Scale is the same for all plots.

Grand averages can be calculated across all subjects (Code Snippet 25). The grand averages can be seen in Figure 13. One thing to keep in mind when doing MEG is that channels will align differently to the head across subject due to fixed positions of the sensor helmet and the different sizes and shapes of subjects' heads. This is in contrast to electroencephalography (EEG), where the EEG-cap is in the same relative place on all subjects. This difference in alignment has the consequence that grand averages should be interpreted with some care. Still the beta rebound is nicely present on all stimulations (Figure 13).

Applying the function *calculate_grand_average_tfr* takes ~8 min.

**Code Snippet 25:**
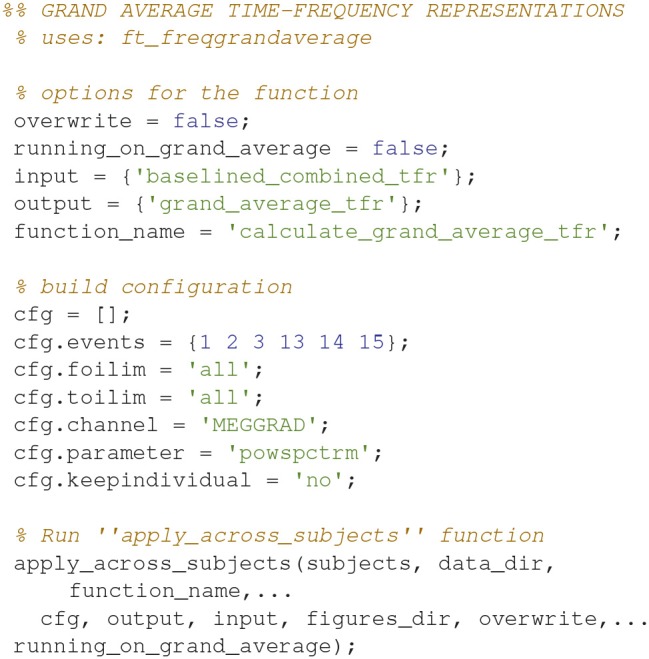
Code for calculating the grand averages for time-frequency representations.

### Grand averages, beamformer

**Figure 14:**
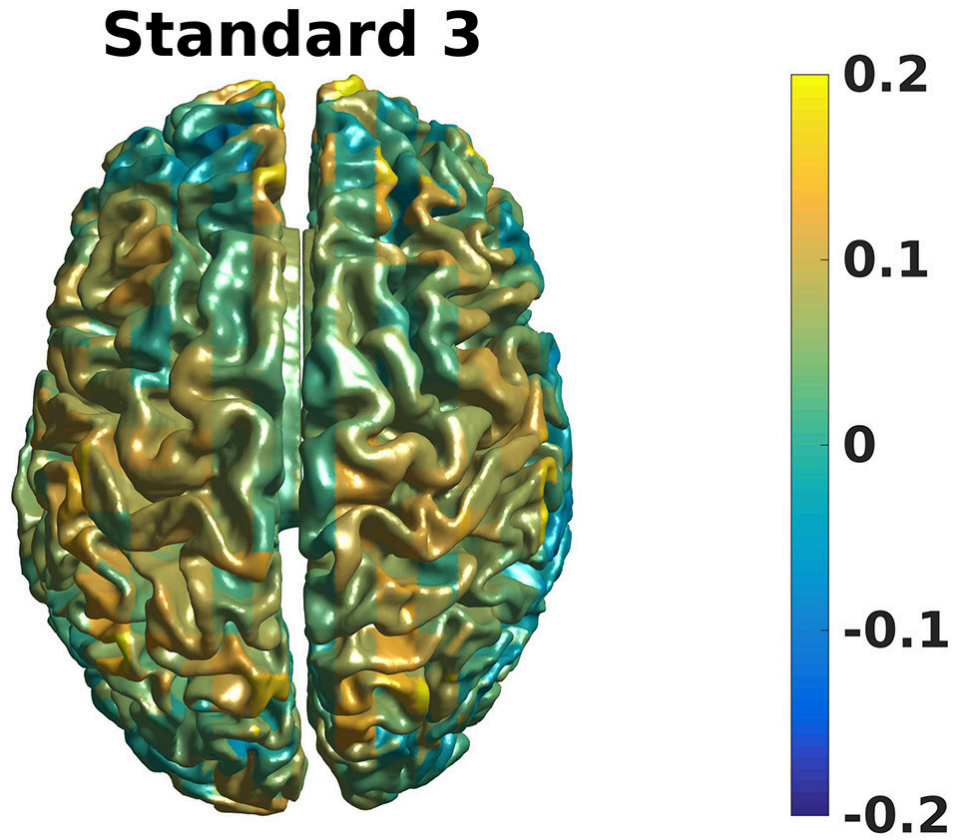
Grand average beamformer contrast. Color shows where there is more/less power for Standard 3 when compared to Non-Stimulation. (0 means equal power, and 0.2 means 20% more power).

Grand averages can also be calculated across subjects since we used warped grids for the leadfield (Code Snippet 26). An example grand average can be seen in Figure 14 (note that interpolation is done before plotting on the common surface).

Applying the function *calculate_grand_average_beamformer* takes ~10 min.

**Code Snippet 26:**
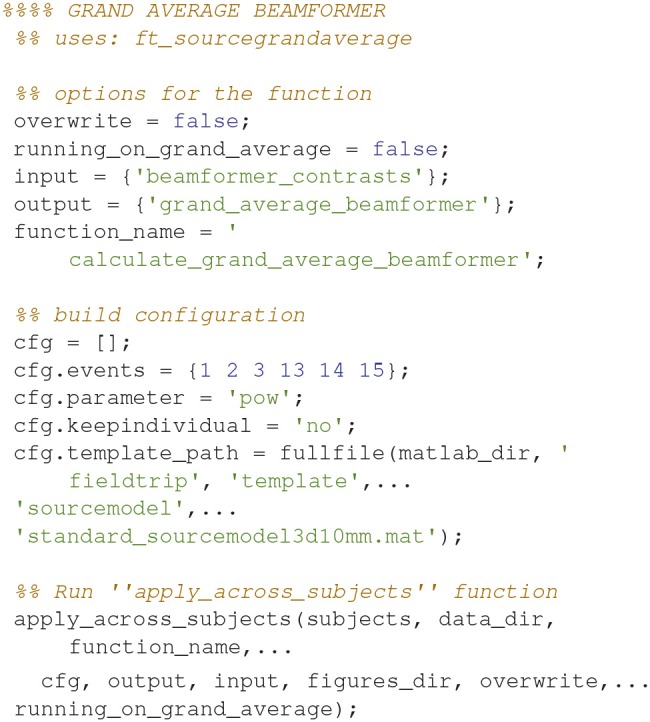
Code for calculating the grand averages for the beamformer source reconstructions.

### Grand averages, beamformer interpolation

To plot statistically thresholded grand averages, it is necessary to interpolate the grand averaged data onto a common template (Code Snippet 27).

Applying the function *interpolate_grand_average_beamformer* takes ~10 s.

**Code Snippet 27:**
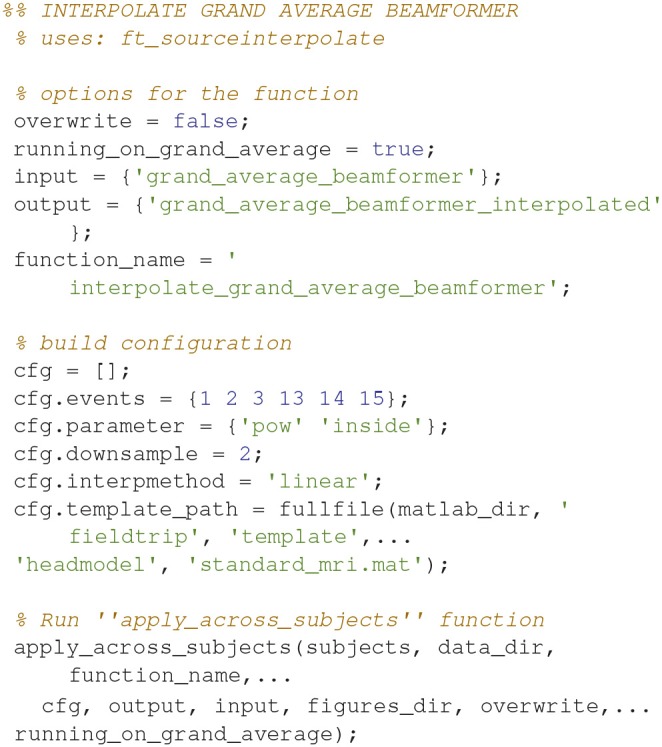
Code for interpolating the beamformer source reconstructions onto a common template.

## Statistics—source space

The statistics script is dependent on the functions in the *statistics_functions* folder (Table 11). Note that *running_on_grand_average* and *apply_across subjects* are also used here, as they are in the *grand_averages* script. Mass-univariate tests can be run on both time-frequency representations and on the beamformer source reconstructions. In the examples, no corrections are done for multiple comparisons. The code can be easily amended to do more advanced statistical testing, such as cluster analysis (Maris and Oostenveld, 2007). See *ft_freqstatistics* and *ft_sourcestatistics* for instructions on how to perform these.

**Table 11:** Functions related to source space operations in the *statistics_functions* folder and a brief description of what their purposes are.

**File Names**	**Description**
*statistics_beamformer.m*	Do statistics on the beamformer source reconstructions
*interpolate_statistics_beamformer.m*	Interpolate the statistics from the beamformer source reconstructions onto a common template

*Functions are put in the order that they are meant to be applied*.

### Statistics, beamformer

Statistical significance can be assessed for the source reconstructed activity (Code Snippet 28) in a manner similar to how it was done for the sensor space activity (Code Snippet 21).

Applying the function *statistics_beamformer* takes ~9 min.

**Code Snippet 28:**
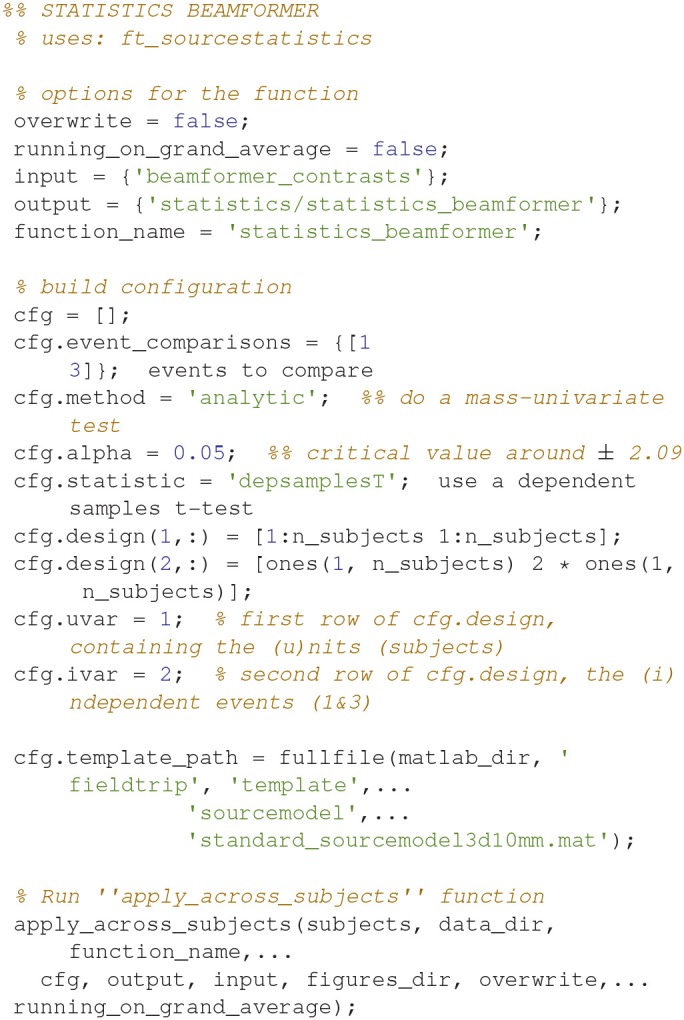
Code for calculating the statistics for the beamformer source reconstructions.

### Interpolate beamformer statistics

**Figure 15:**
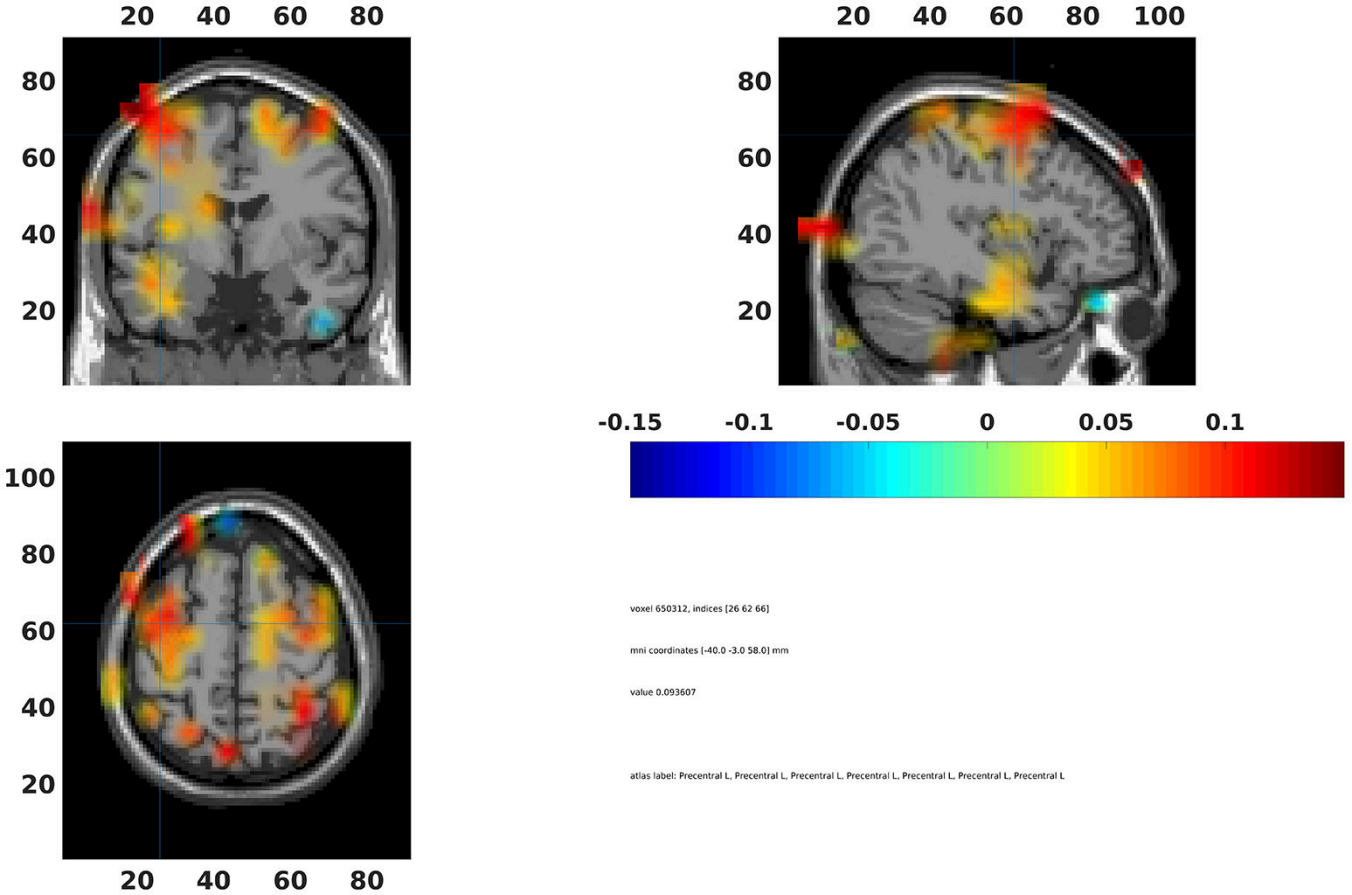
Grand average beamformer interpolated onto a common template and non-significant voxels assigned no color. Colors indicate difference between Standard 1 and Standard 3. The cross-hair is centered on the contralateral motor cortex. Ipsilateral activation is also seen in the motor cortex.

The statistical values can also be interpolated onto a common template (Code Snippet 29). In Figure 15 a source plot can be seen where the non-significant changes have been masked. The differences in the beta rebound between novel and repeated stimulations was localized to the somatosensory cortex, the motor cortex, the supplementary motor area and the insula. These results fit well with findings in the literature (Cheyne, 2013).

Applying the function *interpolate_statistics_beamformer* takes ~5 s.

**Code Snippet 29:**
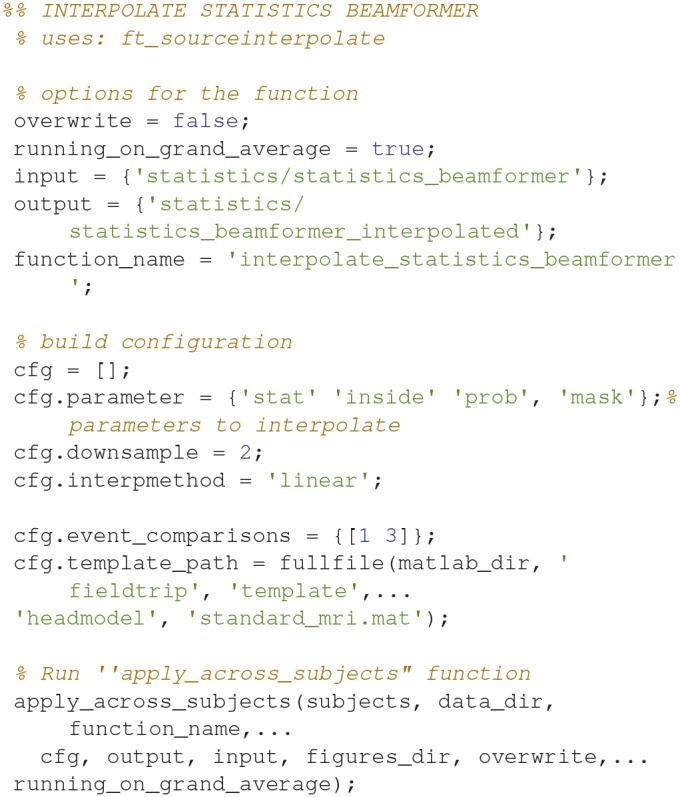
Code for interpolating the beamformer statistics onto a common template.

### Summary of analysis

On the sensor level we found the differences in the beta rebound bilaterally (Figure 10) across the central sensors, but with maximal power contralaterally (Figures 15). In the source domain the differences in the beta rebound between novel and repeated stimulations was localized to the somatosensory cortex, the motor cortex, the supplementary motor area, and the insula. These results fit well with findings in the literature (Gaetz and Cheyne, 2006; Gaetz et al., 2010; Cheyne, 2013).

## Discussion

The presented pipeline allows for covering all steps involved in a FieldTrip pipeline focussing on induced responses and the localization of their neural origin. Furthermore, it also supplies a very flexible framework that users should be able to extend the to meet any further needs that the user may have. For the functions that rely on FieldTrip functions, a user can easily change and add parameters in the normal FieldTrip way by adding and changing fields in the configuration (*cfg*) structures. To change the frequency to be reconstructed, for example, one can change the *foilim* field when making the Fourier transform (Code Snippet 23). It is also easy to include further steps in the analysis such as calculating connectivity, doing other kinds of source reconstructions such as Minimum Norm Estimates (Hämäläinen and Ilmoniemi, 1994).

### Comparison with other type of pipelines

The presented pipeline is especially use for extracting and imaging neural activity that is not phase-locked to any presented stimulation. When phase-locked activity is of interest, such as the time-locked activity depicted in Figure 6, there are other strategies that may work better, such as dipole fitting (Mauguière et al., 1997; Hari and Puce, 2017) or distributed source reconstructions such as the Minimum Norm Estimates (Hämäläinen and Ilmoniemi, 1994) mentioned above. These strategies work especially well for primary sensory responses that are often tightly phase-locked both within and across subjects. Also when there are distal coherent sources in the brain, beamformer might fail as discussed below.

## Possible pitfalls and limitations

A major assumption of beamformer approaches is that it is assumed that no two extended sources are correlated with one another on the extent of square millimeters (van Veen and Buckley, 1988; Hillebrand and Barnes, 2005). Linearly correlated sources cannot be imaged faithfully with beamforming approaches. (van Veen et al., 1997) showed that for two highly correlated sources, a beamforming approach reconstructed a single source in between the two sources. Hillebrand and Barnes (2005) argue that beamforming approaches generally work well, however, because neuronal processes are generally locally coherent but globally incoherent. A good example, however, of when this assumption is not met is when auditory stimulation is presented binaurally. The neuronal activity in the two auditory cortices will be coherent because they are phase-locked to the presentation of the stimulus. The paradigm used in this protocol article is likely to meet the assumption of uncorrelated sources since stimulation is presented unilaterally.

What may also be problematic with sensor-space analyses of induced responses is that the calculation of the grand average of sensors (as seen in e.g., Figure 14) rests on the assumption that the sensors measure the same neural activity across subjects. This is not likely to be the case since head shapes vary considerably between subjects. A possible strategy is to transform the head position of each subject to a position shared between subjects such as is possible with the MaxFilter software from Elekta. Another strategy employed here, is to perform the key analyses related to corroborating one's hypothesis in source space thereby eliminating the problem of sensors not measuring the same neural activity across subjects. The problem is not completely eliminated by doing the key analyses in source space, though, since there is a multitude of different time- and frequency-ranges one could choose to source reconstruct with a beamformer approach. Performing all possible source reconstructions for a given data set would cause a massive multiple comparisons problem, therefore statistics on the sensor space data can be used to constrain the number of time- and frequency-ranges one runs one's source reconstructions on. Constraining the number of source reconstructions in this manner, however, makes it clear that the analysis of induced responses is still dependent on the assumption of the sensors measuring the same neural activity across subjects. As long as this assumption is partially met, one might still find robust and statistically significant responses, such as the beta rebound effect found here.

## Author contributions

The author confirms being the sole contributor of this work and approved it for publication.

### Conflict of interest statement

The author declares that the research was conducted in the absence of any commercial or financial relationships that could be construed as a potential conflict of interest.
